# The actions of methotrexate on endothelial cells are dependent on the shear stress-induced regulation of one carbon metabolism

**DOI:** 10.3389/fimmu.2023.1209490

**Published:** 2023-06-30

**Authors:** Marie B. Lang, Kit-Yi Leung, Nicholas D.E. Greene, Kerri M. Malone, Gaye Saginc, Anna M. Randi, Allan Kiprianos, Robert T. Maughan, Charis Pericleous, Justin C. Mason

**Affiliations:** ^1^ National Heart and Lung Institute, Imperial College London, London, United Kingdom; ^2^ Developmental Biology & Cancer Department, UCL Great Ormond Street Institute of Child Health, University College London, London, United Kingdom; ^3^ European Bioinformatics Institute, Cambridge, United Kingdom

**Keywords:** methotrexate, endothelial cells, one carbon metabolism, cardiovascular disease, shear stress

## Abstract

**Objectives:**

The disease-modifying anti-rheumatic drug methotrexate (MTX) is recognized to reduce cardiovascular risk in patients with systemic inflammatory diseases. However, the molecular basis for these cardioprotective effects remains incompletely understood. This study evaluated the actions of low-dose MTX on the vascular endothelium.

**Methods:**

Human endothelial cells (EC) were studied under *in vitro* conditions relevant to inflammatory arthritis. These included culture in a pro-inflammatory microenvironment and exposure to fluid shear stress (FSS) using a parallel plate model. Respectively treated cells were analyzed by RNA sequencing and quantitative real-time PCR for gene expression, by immunoblotting for protein expression, by phosphokinase activity arrays, by flow cytometry for cell cycle analyses and by mass spectrometry to assess folate metabolite levels.

**Results:**

In static conditions, MTX was efficiently taken up by EC and caused cell cycle arrest concurrent with modulation of cell signaling pathways. These responses were reversed by folinic acid (FA), suggesting that OCM is a predominant target of MTX. Under FSS, MTX did not affect cell proliferation or pro-inflammatory gene expression. Exposure to FSS downregulated endothelial one carbon metabolism (OCM) as evidenced by decreased expression of key OCM genes and metabolites.

**Conclusion:**

We found that FSS significantly downregulated OCM and thereby rendered EC less susceptible to the effects of MTX treatment. The impact of shear stress on OCM suggested that MTX does not directly modulate endothelial function. The cardioprotective actions of MTX likely reflect direct actions on inflammatory cells and indirect benefit on the vascular endothelium.

## 1 Introduction

It has long been appreciated that patients with chronic systemic inflammatory diseases, including rheumatoid arthritis (RA), have an increased risk of cardiovascular disease (CVD) (1). Patients with RA display impaired endothelial function (2, 3), which is known to promote atherogenesis and is likely accelerated by the pro-inflammatory environment present in these patients (4). Low-dose methotrexate (MTX) up to 25mg/week is a highly effective disease-modifying anti-rheumatic drug (DMARD) used for the treatment of chronic systemic inflammatory diseases. In those with inflammatory arthritis, MTX use is associated with a reduced risk of composite CVD events by up to 28% (5–7) and reduced incidence of hospital admissions for heart failure by 57% (7). However, the molecular mechanisms underpinning these cardioprotective actions remain to be fully determined and are most likely multifactorial in nature (1, 8).

By design, MTX is a folate analog that is taken up into cells by the reduced folate carrier (RFC), polyglutamated intracellularly and in turn inhibits folate one carbon metabolism (OCM) (8, 9). This metabolic pathway is essential for driving embryogenesis, hematopoiesis, immune cell activation (10), cancer and potentially CVD (9, 11). Atherogenesis is driven by lipid deposition into the arterial wall and reactive inflammatory responses mediated by multiple cell types, including monocytes, T-cells and endothelial cells (EC) (12). By inhibiting dihydrofolate reductase (DHFR) and thymidylate synthase (TYMS), two central OCM enzymes, MTX attenuates inflammatory cell proliferation (13), induces apoptosis in activated T-cells (14) and inhibits nuclear factor ‘kappa-light-chain-enhancer’ of activated B-cells (NFκB) signaling in T-cells by limiting tetrahydrobiopterin (BH4) generation (15). In macrophages, MTX attenuates cytokine-induced cytokine production and NFκB signaling and increases A20 expression (16). In addition, MTX also inhibits the OCM-associated enzyme aminoimidazole-4-carboxamide ribonucleotide (AICAR) transformylase (ATIC) (17), which subsequently leads to enhanced adenosine release and reduced leukocyte accumulation in a murine air pouch model of inflammation (18, 19).

Beyond its immunomodulatory actions, recent clinical studies have addressed the question whether the drug also targets vascular EC. MTX improved endothelial function in patients with inflammatory arthritides, alone or in combination with tumor necrosis factor (TNF)α blockade (20) or other DMARD (21). While MTX exerts potent anti-inflammatory effects in immune cells by OCM inhibition, its effects on EC remain unclear, partly due to the pathway’s poorly defined role in EC. MTX is reported to increase endothelial adenosine release (18), to activate an AMP-activated kinase (AMPK)-CREB-dependent vasculoprotective pathway (22) and reduce the expression of cell adhesion molecules (CAM) (23). In contrast, other reports have shown that exposure to MTX inhibits endothelial cell proliferation (24), increases pro-inflammatory cytokine release in an endothelial-epithelial cell line (25) and predisposes EC to increased superoxide generation and reduced nitric oxide (NO) synthesis by uncoupling endothelial nitric oxide synthase (eNOS) *in vitro* (26). These studies emphasize the need to better understand the function of OCM in EC and the effects of its inhibition by MTX.

Endothelial cells are naturally exposed to flowing blood, which exerts fluid shear stress (FSS). FSS is an important regulator of endothelial function and also atherogenesis by inducing atheroprotective and atheroprone endothelial phenotypes (27). However, most *in vitro* studies have relied on static EC cultures, which do not sufficiently represent these different endothelial states. To address whether anti-inflammatory effects on the endothelium contribute to the drug’s vasculoprotective actions, we explored the molecular effects of MTX on human vascular EC cultured in a pro-inflammatory microenvironment and exposed to FSS, conditions relevant to inflammatory arthritis and the risk of disease-associated cardiovascular events.

## 2 Materials and methods

### 2.1 Cell culture and treatments

Human umbilical vein (HUVEC) and aortic endothelial cells (HAEC; Promocell) were cultured in Endothelial Growth Medium (EGM)-2 (Promocell) at 37°C and 5% CO_2_. Cells were used at passages 3-5. Cell treatments were performed in M199 (Sigma) supplemented with 10%FCS, 100µg/mL penicillin/streptomycin, 2mM glutamine and 15µg/mL ECGS (Millipore). Cell treatments were performed with MTX (100nM; Hospira, UK), TNFα (0.1ng/ml; R&D Systems) and folinic acid (500nM; Sigma).

### 2.2 Shear stress

Shear stress experiments were performed using a commercially available shear stress system (Quad fluidic unit, Ibidi) according to the manufacturer’s instructions. Cells were seeded onto 1% gelatin-coated μ-slides I 0.4 Luer (Ibidi) and exposed to continuous high LSS (H-LSS; 20dyn/cm^2^), low LSS (L-LSS; 5dyn/cm^2^) or OSS (±5dyn/cm^2^, 2Hz) for 48-96h using a parallel flow system (Ibidi). Cells were pre-conditioned with shear stress for 48h before pharmacologic treatment. Due to the experimental set-up used, MTX treatment was only performed under H-LSS and OSS conditions. Static controls were grown on 6-well plates in parallel to the shear stress experiments.

### 2.3 RNA isolation and cDNA synthesis

Cells were washed twice with PBS and lysed in 350μL RNeasy Lysis Buffer (RLT; Qiagen) supplemented with ß–mercaptoethanol (10μL/mL). After harvesting the lysate, RNA was isolated using the RNeasy Mini Kit (Qiagen) following the manufacturer’s instructions. RNA concentrations were measured by UV spectrophotometry using a NanoDrop (Thermo Scientific). RNA was mixed with Quanta qScript cDNA mix (Quanta Biosciences) and water. The reaction was run for 5min at 22°C, 30min at 42°C and 5min at 85°C.

### 2.4 Quantitative real-time polymerase chain reaction

A master mix of IQ SYBR Green Supermix (Bio-Rad), primer mix containing the forward and reverse primers (5μM each) and water was prepared. cDNA was added to the master mix. The PCR protocol included a denaturation step of 95°C for 3min and 40 cycles of 95°C for 10sec and 58°C for 45sec. Real-time PCR reactions were performed using a Bio-Rad CFX96 thermocycler. Target gene expression was normalized to the expression of glyceraldehyde 3-phosphate dehydrogenase (GAPDH). The results were analyzed using the Bio-Rad CFX Manager Software version 2.0 (Bio-Rad) and comparative cycle threshold method (ΔΔCt). Oligonucleotide sequences of the primers used are listed in **Table 1**.

**Table 1:** Oligonucleotide sequences used for qPCR analyses.

Primer	Oligonucleotide sequence 5' to 3'
ATIC fw	CGTGATAAGCCCGGAAACAG
ATIC rev	AGAGACACTAAATAAGGCGAGC
DHFR fw	CACAACCTCTTCAGTAGAAGGTAA
DHFR rev	CTGCCACCAACTATCCAGAC
FPGS fw	ATGGAGTACCAGGATGCCGT
FPGS rev	GGCTTCCAACTGTGTCTGAG
GAPDH fw	CAA CAG CCT CAA GAT CAT
GAPDH rev	GAG TCC TTC CAC GAT ACC
GART fw	ACCCGGTGTCGGTTTCATT
GART rev	CAGCGTATGTTCCCTTCCT
GGH fw	GCATCCAGAGAAAGCACCTT
GGH rev	TGGTTGTTTTTCCGAGCTTCA
KLF2 fw	GCA AGA CCT ACA CCAAGA GTT CG
KLF2 rev	CAT GTG CCG TTT CAT GTG C
MTHFD1 fw	GCTGAAGTCTACACGAAGCA
MTHFD1 rev	CAGGCATTGTGCTCATCGTT
MTHFDIL fw	GGGACTCCATCGTCAGAGAA
MTHFD1L rev	TCACCTGCCTGGATAATTGC
MTHFD2 fw	CTGAGTGTGATCCTGGTTGG
MTHFD2 rev	GTCTCACTGTTGATTCCCACA
SELE fw	TACCTACCTGTGAAGCTCCC
SELE rev	GCTTTCCGTAAGCATTTCCG
SHMT2 fw	CTTCTCTTTGTTTTGGGCGG
SHMT2 rev	GCAGAAGTTCTCTGAGGCAA
SLC19A1 fw	TGCTACCTTTGCTTCTACGG
SLC19A1 rev	TGATCTCGTTCGTGACCTGC
THBD fw	ACGACTGCTTCGCGCTCTACCC
THBD rev	CACCGAGGAGCGCACTGTCATTA
TYMS fw	TGGTTTATCAAGGGATCCACAA
TYMS rev	CAGTTGGTCAACTCCCTGTC
VCAM1 fw	CTACGCTGACAATGAATC
VCAM1 rev	GCAACTGAACACTTGAC

### 2.5 qPCR arrays

Samples were run on pre-designed qPCR arrays with primers against 84 targets important in endothelial biology (Qiagen). The full list of targets is shown in **Table 2**. RNA isolation was carried out as stated above. cDNA synthesis was performed using the RT2 First Strand Kit according to the manufacturer’s instructions (Qiagen). A mix of cDNA, 2x RT2 SYBR Green Mastermix and water were added to the qPCR plates. The PCR program was 1 cycle of 50°C for 2min and 95°C for 10min, followed by 40 cycles of 95°C for 15sec and 60°C for 1min. The reaction was run on a QuantStudio Flex 6 Thermocycler (Applied Biosciences). Target gene expression was normalized to GAPDH. The results were analyzed using the RT2 Profiler PCR data analysis excel spreadsheet supplied by the manufacturer by unpaired T-tests. Genes were excluded if they did not pass the Ct threshold of >35 or melt curve criteria.

**Table 2:** Gene list of EC biology qPCR array (Qiagen).

Gene symbol	Full gene name
ACE	Angiotensin I converting enzyme I
ADAM17	ADAM metallopeptidase domain 17
AGT	Angiotensinogen
AGTRl	Angiotensin II receptor, type 1
ALOX5	Arachidonate 5-lipoxygenase
ANGPT1	Angiopoietin 1
ANXA5	Annexin A5
APOE	Apolipoprotein E
BAX	BCL2-associated X protein
BCL2	B-cell CLL/lymphoma 2
BCL2Ll	BCL2-like 1
CALCA	Calcitonin-related polypeptide aloha
CASP1	Caspase 1, apoptosis-related cysteine peptidase
CASP3	Caspase 3, apoptosis-related cysteine peptidase
CAV1	Caveolin 1, caveolae protein, 22kDa
CCL2	Chemokine (C-C motif) ligand 2
CCL5	Chemokine (C-C motif) ligand 5
CDH5	Cadherin 5, type 2 (vascular endothelium)
CFLAR	CASP8 and FADD-like apoptosis regulator
COL18A1	Collagen, type XVIII, alpha 1
CX3CL1	Chemokine (C-X3-C motif) ligand 1
EDN1	Endothelin 1
EDN2	Endothelin 2
EDNRA	Endothelin receptor type A
ENG	Endoglin
F2R	Coagulation factor II (thrombin) receptor
F3	Coagulation factor III (thromboplastin, tissue factor)
FAS	Fas (TNF receptor superfamily, member 6)
FASLG	Fas ligand (TNF superfamily, member 6)
FGF1	Fibroblast growth factor 1 (acidic)
FGF2	Fibroblast growth factor 2 (basic)
FLT1	Fms-related tyrosine kinase 1 (vascular endothelial growth factor receptor)
FN1	Fibronectin 1
HIF1A	Hypoxia inducible factor 1, alpha subunit
HMOX1	Heme oxygenase (decycling) 1
ICAM1	Intercellular adhesion molecule 1
IL11	Interleukin 11
IL1B	Interleukin 1, beta
IL3	Interleukin 3 (colony-stimulating factor, multiple)
IL6	Interleukin 6 (interferon, beta 2)
IL7	Interleukin 7
ITGA5	Integrin, alpha 5 (fibronectin receptor)
ITGAV	Integrin, alpha V (vitronectin receptor, antigen CD51)
ITGB1	Integrin, beta 1 (fibronectin receptor, antigen CD29)
ITGB3	Integrin, beta 3 (platelet glycoprotein IIIa, antigen CD61)
KDR	Kinase insert domain receptor (VEGFR2)
KIT	V-kit Hardy-Zuckerman 4 feline sarcoma viral oncogene homolog
KLK3	Kallikrein-related peptidase 3
MMP1	Matrix metallopeptidase 1
MMP2	Matrix metallopeptidase 2
MMP9	Matrix metallopeptidase 9
NOS3	Nitric oxide synthase 3
NPPB	Natriuretic peptide B
NPR1	Natriuretic peptide receptor A/guanylate cyclase A
OCLN	Occludin
PDGFRA	Platelet-derived growth factor receptor, alpha polypeptide
PECAMI	Platelet/endothelial cell adhesion molecule
PF4	Platelet factor 4
PGF	Placental growth factor
PLAT	Plasminogen activator, tissue
PLAU	Plasminogen activator, urokinase
PLG	Plasminogen
PROCR	Protein C receptor, endothelial
PTGIS	Prostaglandin 12 (prostacyclin) synthase
PTGS2	Prostaglandin-endoperoxide synthase 2 (COX2)
PTK2	PTK2 protein tyrosine kinase 2
SELE	Selectin E
SELL	Selectin L
SELPLG	Selectin P ligand
SERPINEI	plasminogen activator inhibitor type (PAI) 1)
SOD1	Superoxide dismutase 1, soluble
SPHK1	Sphingosine kinase 1
TEK	TEK tyrosine kinase, endothelial
TFPI	Tissue factor pathway inhibitor
TGFB1	Transforming growth factor, beta 1
THBD	Thrombomodulin
THBS1	Thrombospondin 1
TIMP1	TIMP metallopeptidase inhibitor I
TNF	Tumor necrosis factor
TNFSF10	Tumor necrosis factor (ligand) superfamily, member 10
TYMP	Thymidine phosphorylase
VCAM1	Vascular cell adhesion molecule 1
VEGFA	Vascular endothelial growth factor A
VWF	Von Willebrand factor
ACTB	Actin, beta
B2M	Beta-2-microglobulin
GAPDH	Glyceraldehyde-3-phosphate dehydrogenase
HPRT1	Hypoxanthine phosphoribosyltransferase 1
RPLPO	Ribosomal protein, large, PO

### 2.6 Cell cycle analysis

Cells were harvested by trypsinization, washed twice in 1xPBS and fixed in ice-cold 70% ethanol overnight at 4°C. Fixed cells were washed twice in 1xPBS and stained with a permeabilization buffer containing PBS, 0.1% Triton X-100 (Sigma), 0.05M EDTA (Sigma), propidium iodide (50μg/ml; Sigma) and RNAse A (400μg/ml; Sigma) for 30min at 37°C. Cells were filtered through a 50μm cell strainer (Sysmex) and analyzed by flow cytometry on a CyAN ADP flow cytometer (Beckman Coulter), counting at least 10000 events per sample. For cell cycle analyses of EC exposed to shear stress, 5000 events were counted per sample. Data was analyzed using the FlowJo software.

### 2.7 Apoptosis assay

Cells were seeded overnight and treated with media (control), MTX (100nM) for 48h or hydrogen peroxide (H_2_O_2_; 200μM) for 3h. Serum starvation was performed for 48h in M199 with 0%FCS. Cells were harvested by trypsinization, washed twice in 1xPBS and stained with Annexin V and propidium iodide (PI) in Annexin V binding buffer for 15min at RT in the dark using the Pacific Blue Annexin V Apoptosis detection kit with PI (BioLegend). Annexin V binding buffer was added to each tube and the cells were filtered through a 50μm cell strainer. The cells were analyzed by flow cytometry counting at least 10000 events per sample.

### 2.8 NFκB reporter luciferase assay

HUVEC were transfected with NFκB Luciferase adenovirus (MOI 200; Vector Biolabs) diluted in M199 medium for 2h at 37°C. The medium was removed and the cells were treated with MTX (100nM) for 48h, after which TNFα (0.1 ng/ml) was added to the cells for 4h. The luciferase activity was measured using the Luciferase Assay System (Promega) according to the manufacturer’s instructions. In brief, the cells were washed with PBS and lysed with 1x cell culture lysis reagent (Promega) for 5min on a shaker. The cell lysate was centrifuged for 1min at 17900g to remove debris. The cell lysate was mixed with Luciferase Assay Reagent (Promega) and luminescence was measured on a plate reader.

### 2.9 Immunoblotting

Cells were lysed in RIPA buffer (Thermo Scientific) or modified cell lysis buffer (28) supplemented with complete protease and phosphatase inhibitors (Roche). Cell lysates were spun for 15min at 17900g and 4°C, and the supernatants were harvested. Protein concentrations were determined using the Pierce BCA Protein Assay Kit (Thermo Fisher) or DC protein assay (BioRad) as per manufacturer’s protocol on a microplate reader (Bio-TEK). The proteins were separated by SDS-PAGE. Membranes were blocked with 5% BSA/TBS-T (Tris-buffered saline (TBS) with 5% w/v BSA; 0.1% Tween-20), followed by incubation with the primary antibodies over night at 4°C (**Table 3**). After washing, the membranes were incubated with the HRP-coupled secondary antibodies in 5% BSA/TBS-T for 1h at RT. The membranes were incubated with ECL chemiluminescence (GE Healthcare) and proteins were detected with Care stream Kodak BioMax light films (Sigma). Alternatively, the blots were probed with the secondary antibody goat anti-mouse IgG Dylight 800 in 5% BSA/TBS-T. The proteins were detected with the Odyssey CLx imaging system (LI-COR Biosciences). Uncropped images of western blots are shown in **Supplementary Figures 10-12.**

**Table 3:** Antibodies used for immunoblot detection.

Antibody	Species	Company (Reference #)	Concentration
p-Akt S473	Rabbit	Cell Signaling (#4060)	1:2000
Akt	Rabbit	Cell Signaling (#4685)	1:2000
Anti-mouse IgG Alcxa Fluor 680	Goat	Invitrogen (#A32729)	1:3000-10000
Anti-rabbit IgG Dylight 800	Goat	Invitrogen (#SA5-35571)	1:3000-10000
DHFR	Rabbit	Abcam (#ab133546)	1:1000
HRP-tagged anti-rabbit IgG	Swine	DAKO (#P0399)	1:5000- 1:40000
HRP-tagged anti-mouse IgG	Goat	DAKO (#P0447)	1:5000 1:40000
MTHFD2	Rabbit	Abcam (#ab151447)	1:1000
p-p38 T180/Y182	Rabbit	Cell Signaling (#4511)	1:5000
p38	Rabbit	Cell Signaling (#8690)	1:5000
Thrombomodulin	Mouse	Santa Cruz (#sc13164)	1:500
a-Tubulin	Mouse	Cell Signaling (#3873)	1:20000
VCAM-1	Rabbit	Cell Signaling (#12367)	1:1000
Vinculin	Rabbit	Cell Signaling (#13901)	1:10000

### 2.10 Phospho-kinase arrays

Cells were lysed with M-PER Mammalian Extraction Buffer (Thermo Fisher) containing Halt Phosphatase Inhibitor Cocktail and Halt Protease Inhibitor Cocktail, EDTA free (Thermo Fisher). Phospho serine-threonine (STK) or tyrosine kinase (PTK) arrays (Pamgene) were performed according to the manufacturer’s instructions. Image and data analyses were performed using the BioNavigator software (Pamgene). The phospho-kinase activity arrays were validated by immunoblot analyses for specific targets.

### 2.11 Folate metabolites

HUVEC were subjected to static conditions, H-LSS or OSS for 48h using the parallel plate model, harvested, snap frozen and stored at -80°C. Alternatively, HUVEC were treated with MTX for 48h under static conditions. Folate metabolites were measured in cell lysates using an established UPLC-MS/MS protocol (29). Briefly, cell pellets were resuspended in a buffer of 20mM ammonia acetate (Sigma), 0.1% ascorbic acid (Sigma), 0.1% citric acid (Sigma) and 100mM DTT (Sigma) at pH 7. After sonication, proteins were precipitated by mixing with acetonitrile (2x sample volume; Thermo Fisher) and centrifugation for 15min at 12000g and 4°C. Supernatants were lyophilized and stored at -80°C. For analysis, lyophilized samples were diluted in ultrapure water, centrifuged for 5min at 12000g and 4°C and analyzed by reversed-phase UPLC (Acquity UPLC BEH C18 column; Waters). The solvents used for UPLC analyses were: Buffer A: 5% methanol (Thermo Fisher), 95% water and 5mM dimethylhexylamine (Fluka) at pH 8.0; Buffer B: 100% methanol, 5mM dimethylhexylamine. After pre-equilibration of the column with 95% Buffer A: 5% Buffer B, the UPLC was run on 95% Buffer A: 5% Buffer B for 1min, followed by a gradient of 5-60% Buffer B over 9min and then 100% Buffer B for 6min before re-equilibration for 4min. The flow rate was 500nl/min. The UPLC was coupled to a XEVO-TQS mass spectrometer (Waters) operating in negative-ion mode using the following settings: capillary 2.5kV, source temperature 150°C, desolvation temperature 600°C, cone gas flow rate 150 L/h and desolvation gas flow rate 1200 L/h. Folate metabolites were measured by multiple reaction monitoring.

### 2.12 RNA sequencing analysis of endothelial cells

HUVEC were isolated from freshly obtained umbilical cords by collagenase digestion as described previously (30). Umbilical cords were collected with informed consent and approval by Imperial College Healthcare Tissue Bank (reference: VAS_AR_18_027). HAEC were obtained commercially (Promocell). HUVEC and HAEC from four donors each were cultured in EGM-2 until confluent and harvested at passage 4. RNA was isolated as above and strand specific, poly-A enriched libraries were sequenced by a commercial provider (Genewiz) to a target depth of 30 million reads per sample. After read quality assessment (FastQC v0.11.7), mapping and transcript quantification was performed with Salmon (v.0.14.2) (31) using the Human reference transcriptome (Gencode release 35). Gene-level expression was summarised as transcripts per million (TPM). Differential expression (DE) analysis between HAEC and HUVEC samples was conducted using EdgeR (v3.2.4) (32) with a false discovery rate of ≤0.05. Raw sequence data are available from the authors on request only due to ethical and legal considerations. Processed gene expression data is deposited on figshare (https://doi.org/10.6084/m9.figshare.21896961.v1 ).

### 2.13 Data analysis

Data analysis was carried out using the GraphPad Prism 9.0 Software (Graph Pad, USA). Data were analyzed by unpaired T-tests or one-way ANOVA and Sidak test for multiple comparisons and shown as the mean ± SEM. Quantification of immunoblots was performed using the ImageJ software (v2.0). Flow cytometry data was analyzed using the FlowJo software (BD Life Sciences). P-values <0.05 were considered significant.

## 3 Results

### 3.1 Methotrexate treatment causes cell cycle arrest by OCM inhibition in static EC

**Figure 1:**
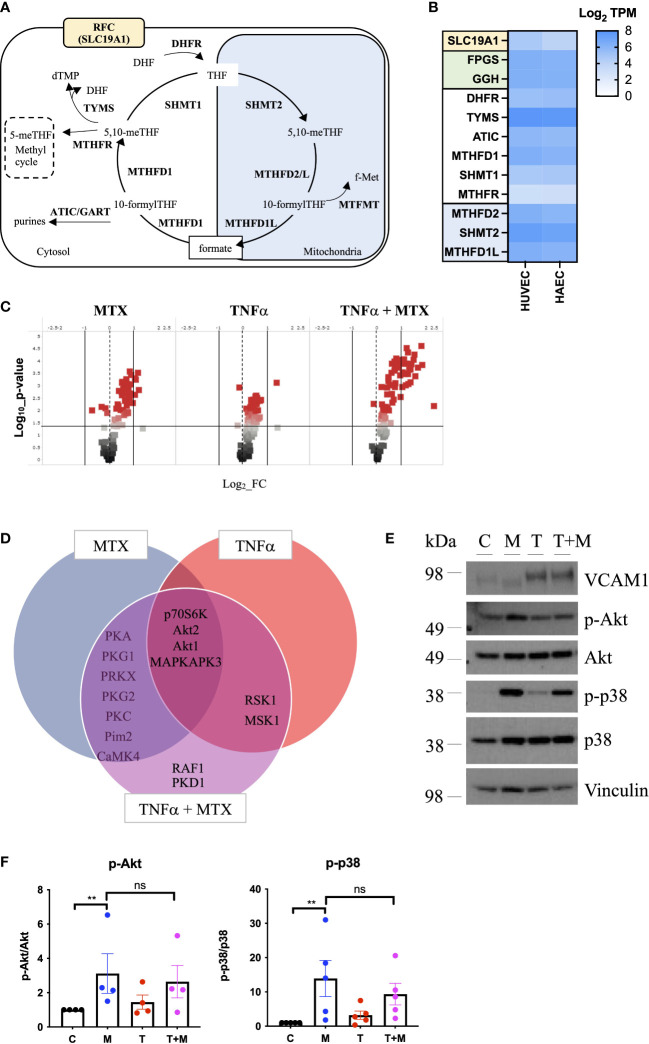
MTX activates cell signaling in static EC. **(A)** Schematic representation of mammalian OCM. The illustration was adapted from (9). **(B)** RNA sequencing analyses of selected transporters and enzymes related to OCM in HUVEC and HAEC cultured under static conditions expressed as Log_2_ transcripts per million (TPM). Proteins involved in cellular folate transport (yellow) and (de-)polyglutamation (green). Enzymes in the cytosolic (white) and mitochondrial (blue) OCM compartment. **(C-F)** HAEC were pre-treated with media (control) or TNFα (0.1ng/ml) for 8h. Media or MTX (100nM) were added to the cells for another 48h (TNFα treatment for total of 56h). **(C)** Volcano plots of differentially phosphorylated peptides in a given condition over control from phospho-kinase arrays (n=3). **(D)** Venn diagram of top kinase hits in the TNFα + MTX co-treatment group. **(E)** Validation of phospho-kinase arrays by immunoblotting of cell lysates from HAEC treated as described above (C, control; M, MTX; T, TNFα; T+M, TNFα + MTX). Representative blots shown (n=4-5). **(F)** Quantification of p38 and Akt phosphorylation levels in HAEC treated as described above (n=4-5). One-way ANOVA and Sidak test for multiple comparisons. Values represent means +/- SEM. ns, not significant. *p<0.05. **p<0.01.

As MTX is processed intracellularly by the folate machinery (8), we first demonstrated basal expression of key OCM genes in MTX-naive EC by comparative analysis of venous and arterial human EC using RNA sequencing (**Figure 1B**). A schematic overview of genes involved in OCM in both the cytosolic and mitochondrial compartments is shown in **Figure 1A**. Subsequent use of an established UPLC-MS protocol (29) suggested measurable uptake of MTX by EC under static conditions in a preliminary experiment (**Supplementary Figure 1**).

To explore the functional effects of MTX on EC, cells were treated under static conditions with MTX (100nM), a concentration achieved in plasma following low-dose therapy (33). At this concentration, MTX treatment did not induce apoptosis in EC (**Supplementary Figure 2**). To understand the effects of MTX on pro-inflammatory signaling, EC were pre-treated for 8h with TNFα, followed by the addition of MTX for 48h to model the pro-inflammatory environment present in patients. EC were analyzed using serine-threonine phosphokinase activity arrays. MTX induced differential phosphorylation of 51 peptides (log2 FC range -0.69 to 1.21; p<0.05, **Figure 1C**), including increased activity of kinases in the PKG, PKA, PKC, Akt and MAPK families (top kinase hits provided in **Supplementary Figure 3**). TNFα treatment altered the phosphorylation of 55 peptides (log2 FC range -0.19 to 1.41; p<0.05), leading to MAPK pathway activation as expected. Co-treatment of TNFα and MTX altered the phosphorylation pattern of 73 peptides (log2 FC range -0.09 to 2.34; p<0.05). Most kinase targets in the co-treatment group were shared with the MTX only group (**Figure 1D**), suggesting that MTX did not inhibit TNFα-induced pro-inflammatory signaling in EC but independently activated endothelial cell signaling pathways. In line with this data, MTX did not inhibit TNFα-mediated activation of NFκB signaling, as demonstrated by a NFκB luciferase reporter, phosphorylation of p65 (S536) or IκBα degradation in EC (**Supplementary Figure 4**).

**Figure 2:**
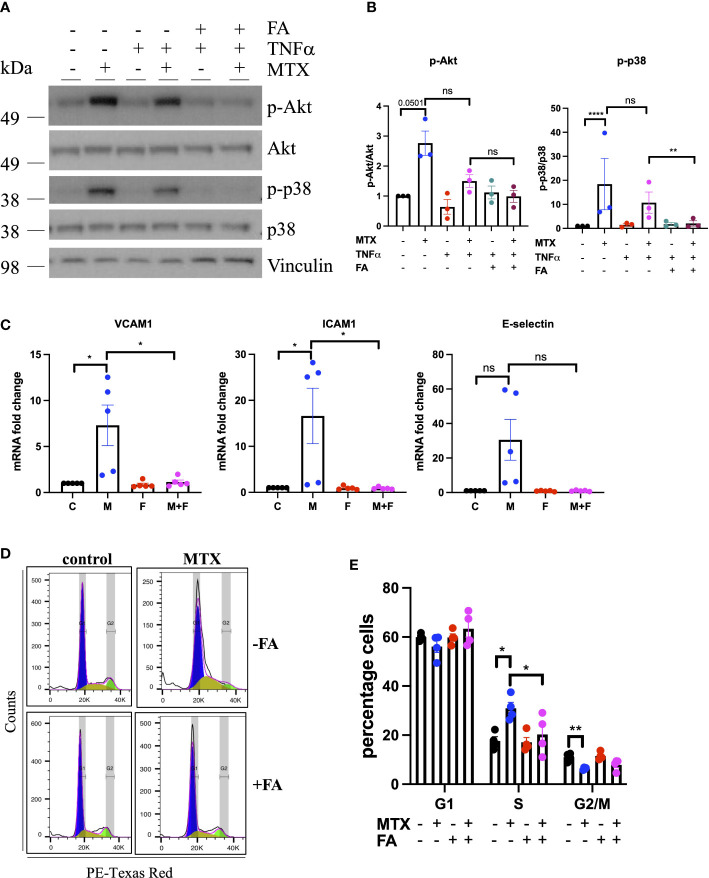
MTX inhibits cell cycle progression in EC by inhibiting OCM. **(A, B)** HUVEC were pre-treated with media (control) or TNFα (0.1ng/ml) for 8h. MTX (100nM) was then added for 48h. After the first 24h of MTX treatment, FA (500nM) was added for 24h. **(A)** Immunoblot analyses of HUVEC treated as outlined. Proteins were detected with antibodies against p-Akt S473, Akt, p-p38 T180/Y182, p38 and Vinculin (n=3). **(B)** Quantification of p38 and Akt phosphorylation in HUVEC treated as described above (n=3). **(C)** Gene expression analyses of HUVEC treated with media (control) or MTX (100nM) for 48h. After the first 24h of MTX treatment, FA (500nM) or vehicle control were added for 24h. Data were normalized to GAPDH and shown as fold change over control (n=5). C, control; M, MTX; F, FA; M+F, MTX+FA. **(D)** Representative flow cytometry profiles of respectively treated cells stained with propidium iodide (PI) and analyzed by flow cytometry (n=4). **(E)** Quantification of cell cycle experiments performed as stated above (n=4). One-way ANOVA and Sidak test for multiple comparisons. Values represent means +/- SEM. ns, not significant. *p<0.05. **p<0.01. ****p<0.0001. Phases of cell cycle: G1, blue; S, yellow, G2/M, green.

Changes in p38 (as a MAPK family target) and Akt phosphorylation were chosen for further validation by immunoblotting as these pathways have been previously associated with roles in EC homeostasis (34). MTX induced the phosphorylation of Akt (S473) 3-fold and p38 MAPK (T180/Y182) up to 14-fold independent of TNFα co-treatment (**Figures 1E, F**). Given that patients with RA receiving MTX are co-prescribed folic acid to minimize side effects, EC were studied in the presence or absence of TNFα, with or without addition of folinic acid (FA) to extend the *in vitro* model (**Figure 2**). Under these conditions, MTX-induced phosphorylation of p38 MAPK (T180/Y182) was interestingly attenuated by the inclusion of FA after 24h, with a similar trend for Akt (S473) (**Figures 2A, B**). FA also reversed the induction of pro-inflammatory cell adhesion molecules (CAM) VCAM1 and ICAM1 mRNA expression by MTX, with a similar effect seen for E-selectin (**Figure 2C**).

Due to the importance of OCM in cell proliferation, the impact of MTX on EC cell cycle progression was investigated, in the presence and absence of FA. In static culture, MTX increased the percentage of cells in the S-phase of the cell cycle (MTX 30.95% *vs.* control 17.6%; p=0.0001), while reducing those in the G2/M phase (MTX 6.35% *vs.* control 11.1%; p<0.0001) (**Figures 2D, E**). Pre-treatment with TNFα alone had no effect on cell cycle progression, nor did it influence the impact of MTX (data not shown). The addition of FA prevented MTX-induced S-phase arrest (MTX 30.95% *vs.* MTX+FA 20.2%). Analysis of EC folate metabolites in a preliminary experiment indicated that MTX may lower the total amount of folates in EC with increased DHF and reduced 5-meTHF and 10-formyl-THF levels (**Supplementary Figure 1**). Taken together, these findings suggested that MTX induced CAM expression and cell cycle arrest in static EC by inhibiting OCM.

### 3.2 MTX does not affect cell signaling or cell cycle progression in EC under FSS

**Figure 3:**
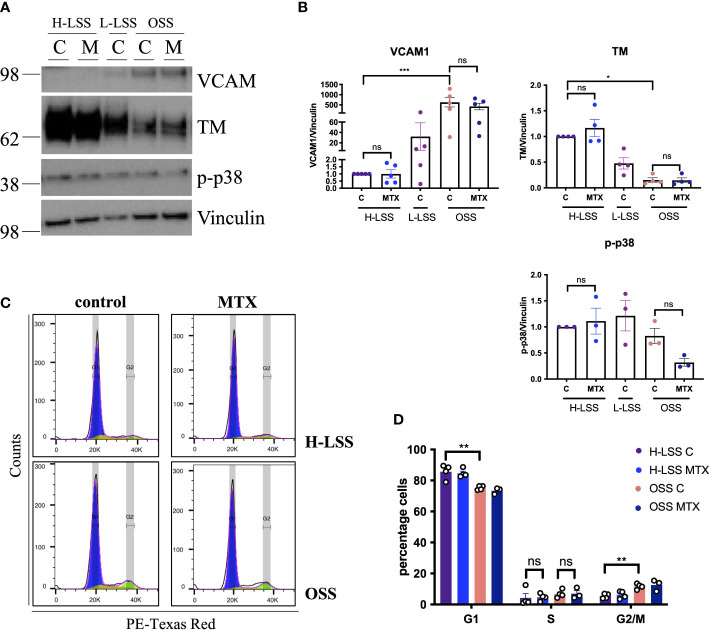
MTX does not affect cell signaling or cell cycle progression in EC under FSS. **(A)** Immunoblot analyses of HAEC pre-conditioned with H-LSS, L-LSS or OSS for 48h. Media (control, C) or MTX (100nM) were added to the cells for a further 48h under H-LSS or OSS (n=3-5). **(B)** Quantification of VCAM1 (n=5), TM (n=4) and p38 phosphorylation levels (n=3) in respectively treated HAEC. Data shown as fold change compared to H-LSS control condition. **(C, D)** HUVEC were pre-conditioned with H-LSS or OSS for 48h, after which cells were treated with media (control, C) or MTX (100nM) for a further 48h under shear stress. **(C)** Representative flow cytometry profiles of respectively treated cells stained with PI and analyzed by flow cytometry (n=3-4). **(D)** Quantification of cell cycle experiments (n=3-4). One-way ANOVA and Sidak test for multiple comparisons. Values represent means +/- SEM. ns, not significant. *p<0.05. **p<0.01. ***p<0.001. Phases of cell cycle: G1, blue; S, yellow, G2/M, green.

*In vivo*, EC are constantly exposed to blood flow; which is not represented by static culture. As shear stress is a fundamental regulator of EC function (27), we aimed to explore the effects of MTX in EC cultured under FSS (**Figure 3**). EC were subjected to static culture, H-LSS, L-LSS or OSS for 48h, followed by MTX treatment for an additional 48h under the respective condition. As controls, the expression levels of thrombomodulin (TM) and VCAM1, two proteins well-known to be regulated by shear stress in EC (27), were measured. EC exposed to OSS expressed lower protein levels of TM (85% reduced; p<0.0052) and higher levels of VCAM1 (625-fold induction; p<0.0002) compared to H-LSS as expected (**Figures 3A, B**). In contrast to static-cultured cells, exposure of EC to prolonged FSS and MTX did not induce p38 phosphorylation. Moreover, MTX did not attenuate OSS-mediated induction of VCAM1 protein expression.

We also performed cell cycle analyses in respectively treated cells under FSS. EC proliferation was increased by OSS when compared to H-LSS, evidenced by the percentage of cells in the G1 (OSS 75.3% *vs.* H-LSS 85.7%; p=0.0003) and G2/M phase (OSS 11.7% *vs.* H-LSS 5.4% p=0.0028) (**Figures 3C, D**). Notably, MTX treatment did not alter the proportion of cells in the G1, S- or G2/M phases in EC under H-LSS or OSS; a finding contrasting with its ability to induce S-phase arrest in EC under static conditions. These results showed that the effects of MTX on EC were dependent on culture conditions and that MTX did not induce p38 phosphorylation or cell cycle arrest in EC under FSS.

### 3.3 MTX does not inhibit OSS-induced pro-inflammatory endothelial activation

OSS induces a pro-inflammatory, pro-proliferative, pro-thrombotic and pro-apoptotic endothelial phenotype (27). Inhibition of these pro-atherogenic effects might contribute to the cardioprotective benefit of MTX treatment in those with RA.

**Figure 4:**
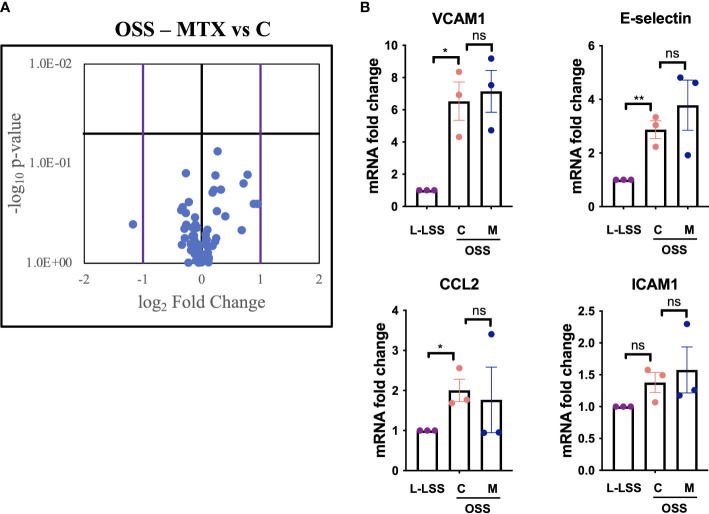
MTX does not inhibit OSS-induced pro-inflammatory endothelial activation. **(A, B)** HAEC were subjected to OSS for 48h. Media (control, C) or MTX (M; 100nM) were added to the cells for another 48h under OSS. As control, HAEC were exposed to low LSS for 96h. Gene expression analyses were performed using qPCR arrays (n=3). **(A)** Volcano plot of endothelial genes in HAEC subjected to OSS with and without MTX. Purple bars indicate fold change >2; horizontal black bar indicates a p-value <0.05. **(B)** Exemplary inflammatory target genes highlighted from gene expression array. Data were analyzed using unpaired T-tests. Values represent means +/- SEM. ns, not significant. *p<0.05. **p<0.01.

To test this, EC were exposed to OSS, treated with MTX and analyzed by a commercially available qPCR array featuring genes relevant to angiogenesis, inflammation, coagulation, platelet activation, apoptosis and vascular tone (**Figure 4**, **Supplementary Figures 5**, **6**). OSS significantly affected the expression of 18 genes (**Supplementary Figures 5A, B**), with VCAM1 and E-selectin induced as expected. OSS induced sphingosine kinase 1 (SPHK1) and reduced VEGFR2 (KDR) and HO1 (HMOX1) expression when compared to L-LSS (**Supplementary Figure 5C**). However, MTX did not significantly alter the expression of any of the genes measured under these conditions (**Supplementary Figure 5C**). Of note, OSS also increased mRNA levels of endoglin (ENG), c-Kit (KIT) and integrin αv (ITGAV).

As shown in **Figure 4**, analysis of key inflammatory genes from the gene expression array including E-selectin, VCAM1, ICAM1 and CCL2 revealed no significant impact of MTX treatment (for full profile see **Supplementary Figure 6**). Thus, evaluation of key endothelial genes regulated by OSS in a parallel-plate flow model suggested that MTX did not exert direct anti-inflammatory effects on the vascular endothelium under these atheroprone conditions.

### 3.4 Endothelial OCM is downregulated by shear stress

To understand why MTX had different effects in EC under FSS, we sought to determine whether FSS altered endothelial OCM. First, a published RNA sequencing dataset of HUVEC cultured under either pulsatile (PS) or oscillatory shear stress (OSS) over 24h was reviewed (35). The expression of transcripts involved in folate transport and poly/de-glutamation were reduced (reduced folate carrier (RFC or SLC19A1) and gamma-glutamyl hydrolase (GGH)), or unaffected (folylpolyglutamate synthase (FPGS)) (**Supplementary Figure 7A**). However, many enzymes in the cytosolic compartment (DHFR, TYMS, methylenetetrahydrofolate dehydrogenase, cyclohydrolase, formyltetrahydrofolate synthetase (MTHFD)1 and serine hydroxymethyltransferase (SHMT)1) were downregulated by both PS and OSS, with DHFR decreased 35% by PS and 50% by OSS (**Supplementary Figure 7B**). In contrast, expression levels of ATIC and methylenetetrahydrofolate reductase (MTHFR), enzymes required for purine metabolism and maintenance of the methylation cycle respectively, were not altered. The mitochondrial enzymes responded differently, with MTHFD2, SHMT2 and methylenetetrahydrofolate dehydrogenase (NADP+ dependent)-1-like (MTHFD1L) transcripts left unaffected or marginally increased by shear stress (**Supplementary Figure 7C**).

**Figure 5:**
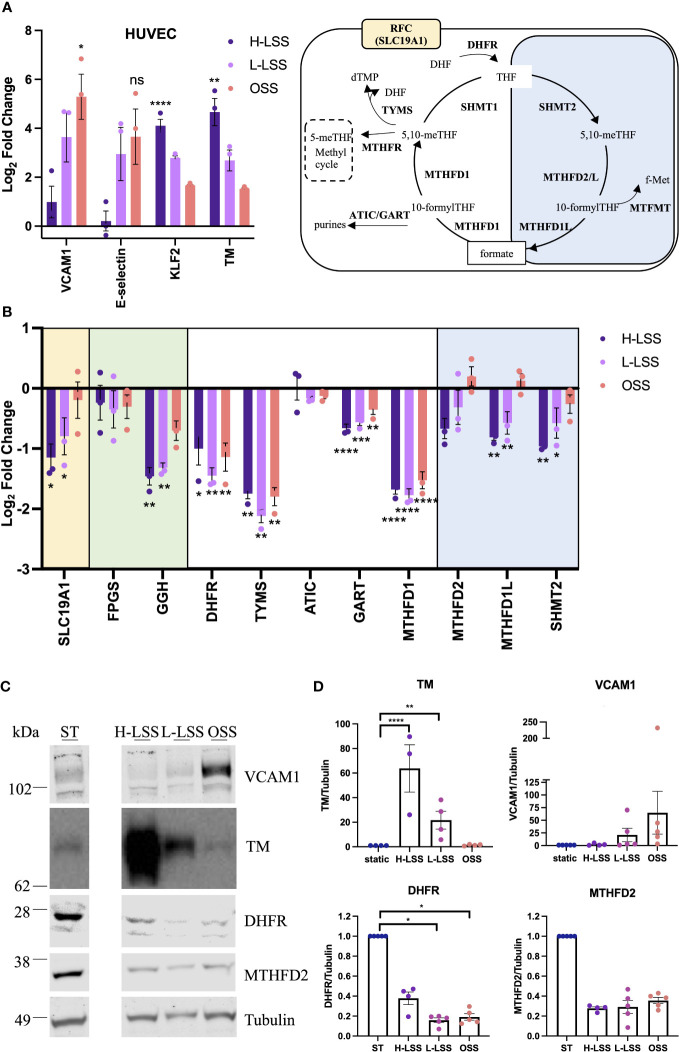
Endothelial OCM is downregulated by shear stress. **(A-D)** HUVEC were subjected to static culture (ST), H-LSS, L-LSS or OSS for 48h. **(A)** Log2 fold changes of the mRNA expression of shear stress-related controls (VCAM1, E-selectin, KLF2 and TM) in HUVEC treated as above by qPCR (n=3). Data shown relative to static condition. **(B)** Log2 fold changes of the expression of selected genes related to OCM in HUVEC treated as above by qPCR (n=3). Data shown relative to static condition. Proteins involved in cellular folate transport (yellow), (de-)polyglutamation (green), enzymatic reactions in the cytosolic (white) or mitochondrial (blue) compartment. **(C)** Immunoblot analyses of respectively treated HUVEC. Proteins were detected with antibodies against VCAM1, TM, DHFR, MTHFD2 and Tubulin (n=3-5). Bands shown from the same blot, with one lane removed. **(D)** Quantification of VCAM1, TM, DHFR and MTHFD2 protein levels in HUVEC treated as above (n=3-5). Data shown relative to static condition. One-way ANOVA and Sidak test for multiple comparisons. Values represent means +/- SEM. ns, not significant. *p<0.05. **p<0.01. ***p<0.001. ****p<0.0001.

Next, expression of selected OCM targets in EC exposed to FSS for 48h were analyzed at the gene and protein level (**Figure 5**). As FSS controls, Krüppel-like factor 2 (KLF2), TM, VCAM1 and E-selectin were measured. H-LSS increased mRNA levels of KLF2 (17.8-fold; p<0.0001) and TM (29-fold; p=0.0061), while OSS increased VCAM1 expression (56.4-fold; p=0.0127) compared to static EC (**Figure 5A**). FSS altered the expression of genes involved in folate transport and poly/de-glutamation, cytosolic and mitochondrial OCM, as measured by qPCR. As seen in **Figure 5B**, in the cytosolic OCM pathway, transcript levels of DHFR (p<0.05), TYMS (p<0.01) and MTHFD1 (p=0.0001) were reduced by both H-LSS and OSS (50-70%) when compared to static conditions. GART was reduced maximally in EC exposed to H-LSS. In contrast, ATIC expression was not altered by shear stress. Mitochondrial MTHFD1L (p<0.01) and SHMT2 (p<0.01) were reduced approximately 50% by H-LSS compared to static cells, while MTHFD2 expression was also reduced without reaching significance. The mitochondrial isoform of FPGS was not affected by FSS, while GGH was significantly reduced by H-LSS. Folate transporter SLC19A1 was reduced by H-LSS (p<0.05) and not affected by OSS, demonstrating differential regulation between H-LSS and OSS (**Figure 5B**). We also confirmed these findings in arterial EC, a cell type relevant to atherosclerosis (**Supplementary Figure 8**). Immunoblotting was performed for selected OCM targets. Protein expression of DHFR was reduced by ≥60% following exposure to FSS (**Figures 5C, D**), in accordance with the mRNA data. MTHFD2 protein expression followed a similar trend but did not reach significance.

**Figure 6:**
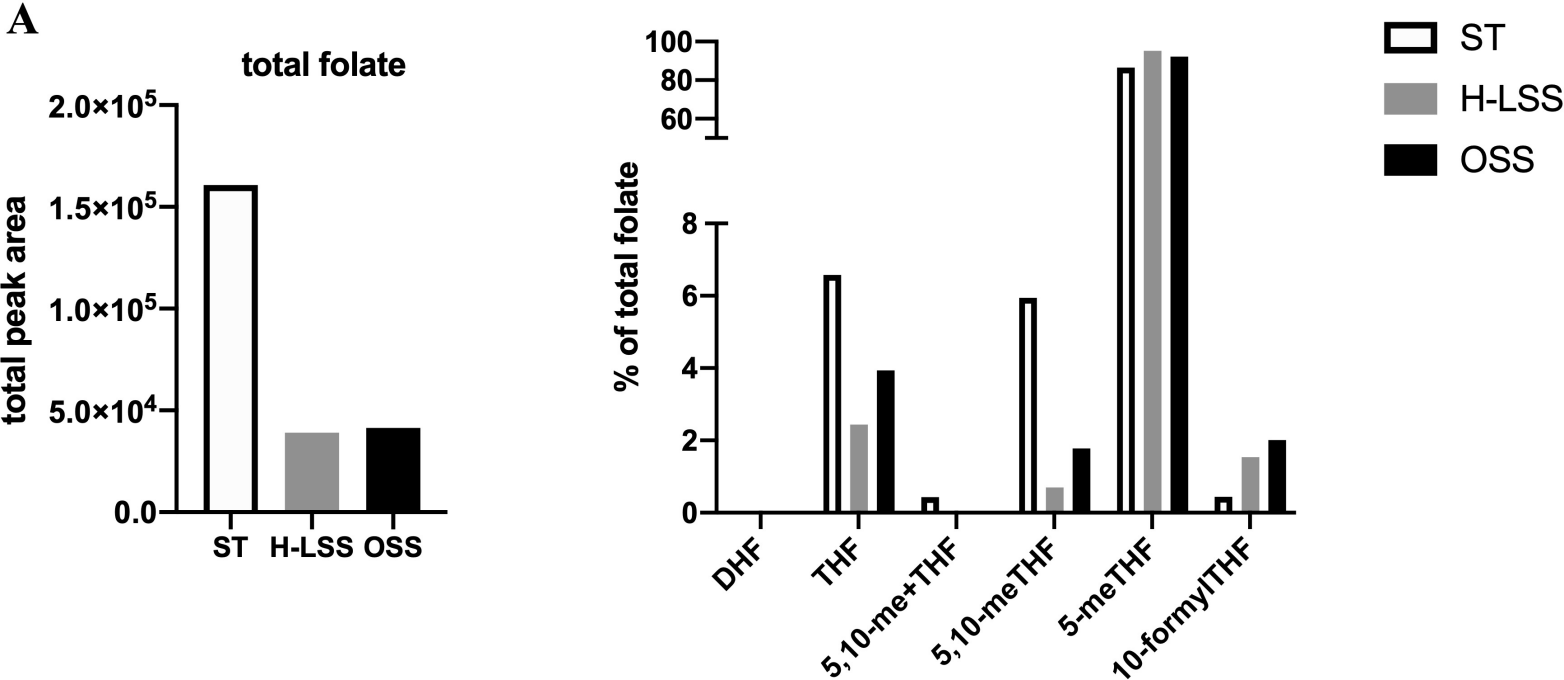
FSS modulates folate metabolite profiles in EC. Folate metabolite profiles of HUVEC subjected to static culture (ST), H-LSS, L-LSS or OSS for 48h and analyzed by UPLC-MS (n=1). Sum of the total peak area of each individual folate metabolite to give the total folate levels in each condition (left). Folate metabolite levels shown as percentage of total folate in each condition (right).

Next, folate metabolites were quantified by UPLC-MS. To compensate for the relatively small amount of lysate generated from the parallel plate flow chambers, lysates from five separate experiments were pooled for analysis (a total of 1.8x10^6^ cells were analyzed). UPLC-MS revealed that the total amount of folate in EC was reduced by up to 75% in EC cultured under FSS compared to static conditions (**Figure 6**). When examining the distribution of each folate as a proportion (%) of total folate, 5-methyl tetrahydrofolate (5-meTHF) levels were not altered by shear stress (**Figure 6**). In contrast, THF, 5,10-me+THF and 5,10-meTHF levels were reduced by H-LSS and OSS when compared to levels in static EC, while dihydrofolate (DHF) was undetectable. 10-formylTHF, required for purine and mitochondrial protein synthesis, was increased by both H-LSS and OSS. Taken together, these experiments demonstrated that endothelial OCM is largely suppressed by FSS. Overall, our findings suggest that although endothelial OCM is important for maintaining EC homeostasis and regulating proliferation under static conditions, the inhibitory effect of FSS on OCM is sufficient to alter the impact of MTX on the vascular endothelium.

## 4 Discussion

**Figure 7:**
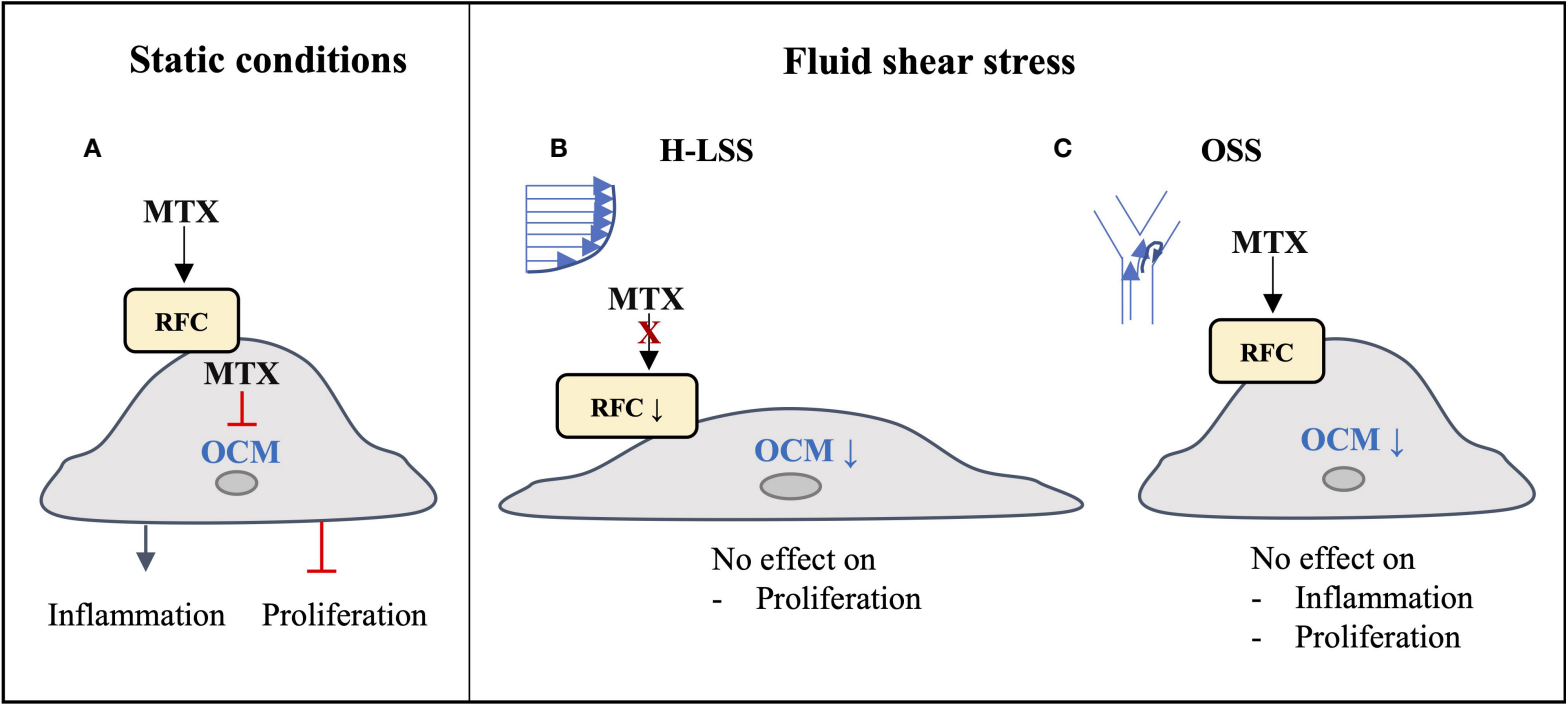
Graphical summary of findings. **(A)** Under static conditions, MTX is taken up into EC and inhibits endothelial OCM, thereby inducing inflammatory activation and inhibiting EC proliferation. **(B)** Under atheroprotective, unidirectional high laminar shear stress (H-LSS) endothelial OCM is downregulated, including the main MTX transporter RFC. MTX does not affect endothelial proliferation under these conditions. **(C)** Disturbed shear stress (oscillatory shear stress; OSS) also downregulates endothelial OCM. Although MTX may be taken up under these conditions, it has no effect on OSS-induced pro-inflammatory activation or proliferation in EC.

The long-acting polyglutamate metabolites of MTX inhibit the folate-dependent enzymes DHFR, TYMS and ATIC, leading to disruption of OCM. Consequences include inhibition of nucleotide synthesis in immune cells and increased extracellular adenosine release, which contribute to the multifactorial immunomodulatory actions of the drug (8). In light of the cardioprotective actions of MTX reported in systemic inflammatory disease (5–7), we investigated whether the drug exerts direct effects in vascular EC subjected to pro-inflammatory activation with cytokines or exposure to FSS. In this report, we show that changes in response to MTX observed in EC cultured under static conditions are not reproduced in EC exposed to prolonged shear stress (**Figure 7**). Anti-inflammatory effects of MTX were not seen in EC conditioned by pro-inflammatory OSS. The demonstration of significant suppression of several members of the endothelial OCM pathway by FSS provides a molecular basis to explain the absence of direct MTX-induced changes on EC cultured under shear stress conditions. Our findings lead us to propose that the beneficial effects of MTX on endothelial function reported in patients with inflammatory disease are predominantly indirect in nature and reflect MTX-induced changes in pro-inflammatory cells and in the inflammatory microenvironment.

Under static culture conditions, metabolic flux through OCM was active in EC. MTX treatment led to phosphorylation of p38 MAPK and Akt, and to cell-cycle arrest. Of note, the impact of MTX was not altered by the presence of TNFα. However, the responses were reversed by FA, implying a key role for OCM. Data from serine-threonine phosphokinase activity arrays suggested that under static culture conditions, the addition of MTX did not significantly impact TNFα signaling in EC. MTX also did not alter NFκB-mediated signaling in EC or reduce TNFα-mediated induction of E-selectin or VCAM1. This contrasts with changes reported in macrophages, where MTX-mediated inhibition of NFκB activity contributes to attenuated IL1 and IL6 release in response to pro-inflammatory mediators (16).

Their location at the blood/tissue interface exposes EC to mechanical forces including FSS. In addition to its impact on constitutive gene expression (27), we have previously shown that FSS alters vascular endothelial responses to exogenous mediators including drugs (36). Initial interrogation of an RNA sequencing dataset (35), and our subsequent experimental work, indicated that in comparison to static-cultured cells, the metabolic flux through OCM was reduced in EC exposed to FSS. Indeed, basal expression of many OCM enzymes and metabolites were downregulated by FSS in EC, with some targets, for example the reduced folate carrier (RFC), showing differential expression under H-LSS and OSS, reduced by the former and maintained under the latter. These results suggested that under physiological conditions, MTX efficacy in EC may be diminished as a consequence of reduced cellular uptake and by downregulation of its prime targets DHFR and TYMS. Previous studies have independently reported decreased DHFR protein (37) and TYMS mRNA (38) levels in EC under LSS. In line with DHFR and TYMS downregulation, MTX did not inhibit cell proliferation in EC subjected to FSS. Furthermore, MTX treatment did not prevent inflammatory activation of EC exposed to prolonged OSS, as evidenced by the inability to reduce OSS-mediated VCAM1 expression. These data are at odds with those of a previous study in which pre-treatment of EC with MTX attenuated the subsequent induction of EC VCAM1 by OSS *via* inhibition of yes-associated protein (YAP) and transcriptional co-activator with PDZ-binding motif (TAZ) (39). However, in the experimental design employed, MTX acts upon EC prior to any effect of OSS on OCM. In contrast, and in light of the fact that EC are continually exposed to shear stress *in vivo*, we chose to pre-condition EC with OSS for 48h prior to the addition of MTX during continued OSS exposure. Under these conditions, MTX was unable to reverse established induction of EC VCAM1 by OSS. Thus, despite expression of RFC being maintained under OSS, MTX did not exert an effect, again implying that reduction of DHFR and TYMS is important. These data suggest that OCM may be dispensable in quiescent EC. Our observation that OCM was suppressed by both atheroprotective and atherogenic shear stress patterns in EC complements prior reports demonstrating regulation of other endothelial metabolic pathways by FSS (40, 41) and implies MTX is unlikely to exert a direct effect. Moreover, these findings are consistent with reports that folate supplementation does not reduce cardiovascular events in patients without systemic inflammatory disease (42–44).

ATIC represents an additional important target of MTX (8). Of note, FSS did not alter endothelial ATIC expression, suggesting that MTX-induced adenosine release may be unaffected under these conditions. Although adenosine levels were not measured in this study, our data suggest that if adenosine was released by EC in response to MTX, it is insufficient to confer an anti-inflammatory effect on EC in this *in vitro* shear stress model. Furthermore, a previous study (35) confirmed that the adenosine receptor ADORA2A, important for mediating the anti-inflammatory action of MTX (45), is also downregulated in EC by FSS (**Supplementary Figure 9**). The lack of a direct impact of MTX on vascular EC exposed to FSS was further illustrated by failure to alter expression of genes included in an endothelial-focused qPCR array. Thus, we propose that if MTX induced endothelial adenosine release under FSS, it would predominantly act on surrounding cells including pro-inflammatory leukocytes. The impact of adenosine on leukocyte trafficking was demonstrated using a murine air-pouch inflammation model, in which the ability of MTX to attenuate leukocyte trafficking was lost in mice deficient in ADORA2A or CD73 (45, 46).

In patients with inflammatory arthritis, tight control of both inflammatory disease activity and traditional CVD risk factors significantly reduces the risk of major CVD events (47, 48). While anti-inflammatory actions of MTX suppressing RA disease activity are important in this regard (6), recent findings also demonstrate improved cardiovascular outcomes independent of the effect of MTX on disease activity (7, 20). While the underlying mechanisms of action remain to be determined, based on our findings we propose that the vascular endothelium is not a direct target of MTX treatment in inflammatory arthritis, rather its actions on neutrophils, macrophages, monocytes, T-cells and fibroblasts indirectly benefit endothelial function and protect against CV events. MTX exerts context-dependent mechanisms of action in these cells, some of which are cell-specific (8). Important amongst these are inhibition of OCM and increased adenosine generation. Indeed, a recent study has reported the key role of OCM in T-cell activation and inflammatory responses (10).

The Cardiovascular Inflammation Reduction Trial (CIRT), a randomized, double-blind placebo-controlled trial of low-dose MTX in those with previous myocardial infarction or multivessel coronary disease and additionally, either type 2 diabetes or metabolic syndrome, illustrates the importance of further defining the mechanisms of action of MTX in the vasculature. In this trial, conducted in patients with a history of CAD but without inflammatory arthritis, no cardioprotective effect of MTX was seen (49). One proposed explanation for the discrepant cardioprotective effects of MTX in those with inflammatory arthritis and those without, is the absence of detectable inflammation (as measured by the high-sensitive C reactive protein assay) in CIRT participants (50). This favors the hypothesis that MTX acts predominantly on inflammatory cells rather than directly on EC. Our data also suggest that suppression of OCM by FSS reduces any potential impact of MTX on endothelial inflammatory responses at atherosclerosis-prone sites exposed to OSS. The actions of MTX on inflammatory cells, including inhibition of OCM, are more likely to have an important role in the setting of chronic inflammatory diseases such as RA. This impact might also extend to those high-risk primary atherosclerosis patients with a C-reactive protein (CRP) level in the higher quartile of the normal range (51), or those with a residual inflammatory risk following optimal statin therapy (52). In this regard, intriguing recent data, from patients presenting with an acute coronary syndrome, suggest that a mildly elevated CRP may represent an important prognostic marker, with the potential to identify and stratify likely responders and non-responders to additional anti-inflammatory strategies such as MTX (53).

The lack of validation *in vivo* is an important limitation of this study. The use of an *in vitro* system was required to help isolate specific effects of MTX on EC in the face of an inflammatory stimulus or conditions of FSS. Future work could now be performed in a murine model using *en face* confocal microscopy of the aorta to interrogate endothelial responses to MTX *in situ*, or by isolating EC for RNA sequencing.

## 5 Conclusion

This is the first report to show that constitutive expression of critical components of OCM in EC is low under physiological shear stress conditions. The suppression by shear stress reversed the impact of MTX seen in static-cultured EC function. These findings do not discredit the beneficial effects of MTX but suggest that the cardioprotective actions of MTX reported in those with systemic inflammatory disease are not mediated by direct effects on the vascular endothelium, but rather by targeting inflammatory responses in other cell types. Further research is now indicated to precisely define context-dependent and cell-specific actions of MTX in the vasculature and to determine its direct and indirect cardioprotective benefits, ultimately aimed at patient stratification and targeted treatment.

## Data availability statement

The datasets presented in this study can be found in online repositories. The names of the repository/repositories and accession number(s) can be found below: https://figshare.com/, https://doi.org/10.6084/m9.figshare.21896961.v1.

## Author contributions

ML, RM, CP, AK, and JM designed the study. ML, KL, RM, and GS performed experimental assays. Data was analyzed by ML, KL, KM, RM, and CP. Manuscript was written and revised by ML, RM, CP, and JM. All authors contributed to the article and approved the submitted version.
