# Supplementary material for: The actions of methotrexate on endothelial cells are dependent on the shear stress-induced regulation of one carbon metabolism
Source: Front Immunol. 2023 Jun 30;14:1209490. doi: 10.3389/fimmu.2023.1209490 (PMC10349526; doi:10.3389/fimmu.2023.1209490)
Supplement: Supplementary file 1 [file Image_1.pdf]

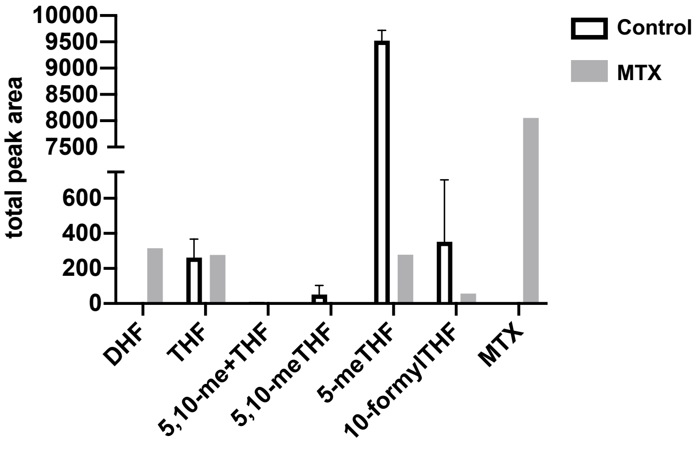

Supplementary Figure 1. Folate metabolite levels in MTX-treated HUVEC. Folate metabolite levels measured in HUVEC treated with MTX (100nM; 48h; 1 technical replicate) compared to control (media); two technical replicates) by UPLC-MS (n=1). Data shown as total peak area of folate metabolites.

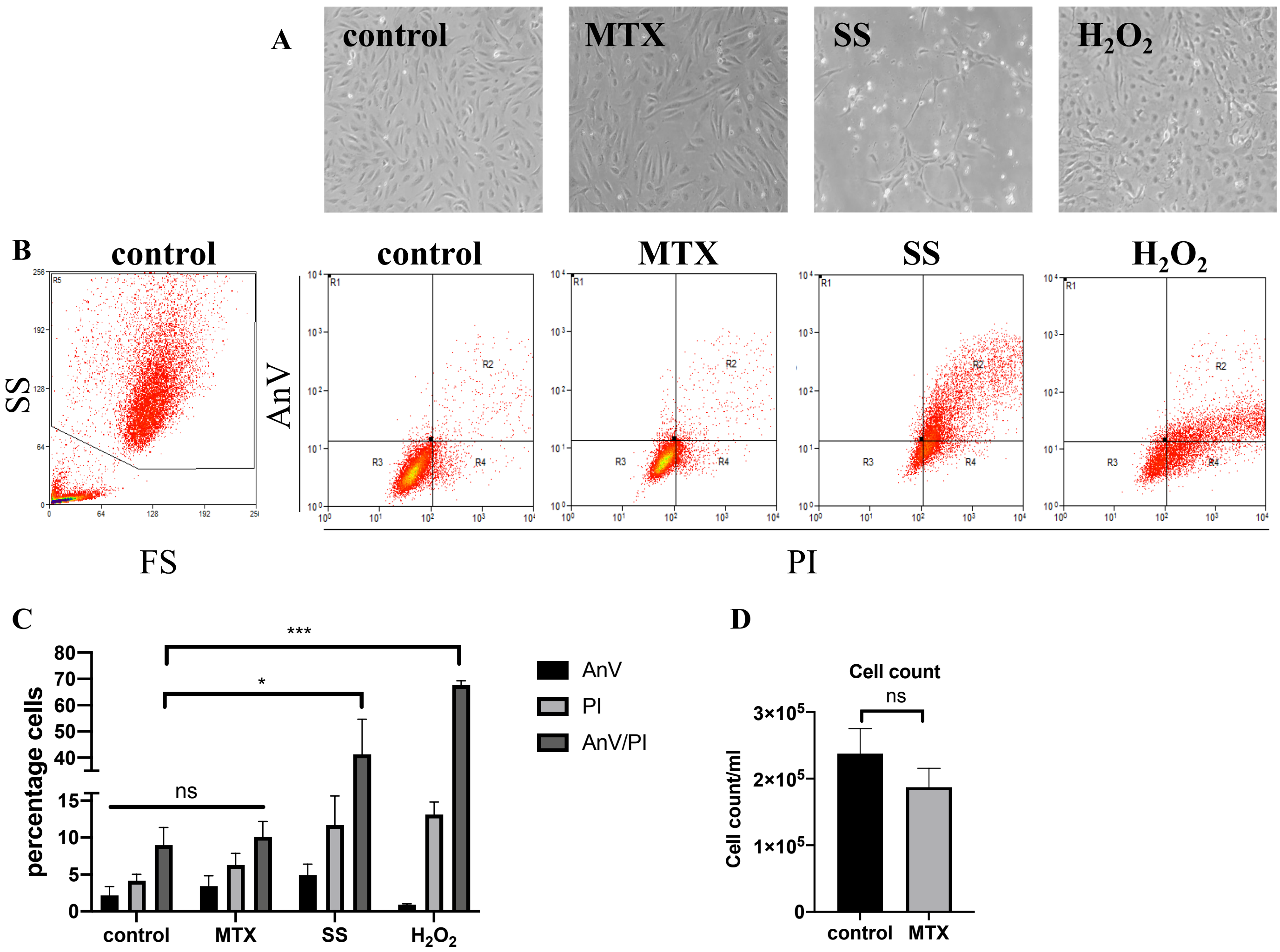

Supplementary Figure 2. MTX does not induce apoptosis in EC. (**A-C**) HAEC were treated with either media containing 10% FCS (control), MTX (100nM; 48h), H_2O_2 (200µM; 3h) or were serum starved in 0% FCS (SS; 48h). (**A**) Representative bright field images (magnification 10x) of respectively treated cells. (**B**) Flow cytometry profiles of cells treated as described above. Cells were stained with annexin V (AnV) and propidium iodide (PI) and analyzed by flow cytometry (n=3). (**C**) Quantification of AnV, PI or double positive HAEC treated as stated above. Data shown as percentages of cells positive for AnV, PI or both (n=3). Data analyzed using a one-way ANOVA and Sidak test for multiple comparisons. (**D**) Cell count of HUVEC grown in the absence (control) or presence of MTX (100nM) for 48h (n=5). Unpaired t-test. Values represent means +/- SEM. ns, not significant. * p<0.05. ** p<0.01. *** p<0.001.

MTX			TNFα			TNFα + MTX		
**Kinase**	**Final score**	**Kinase statistic**	**Kinase**	**Final score**	**Kinase statistic**	**Kinase**	**Final score**	**Kinase statistic**
PKG2	3.7	1.6	ERK5	2.5	1.5	PKA	3.7	2.3
PKG1	3.7	1.6	p38	2.2	1.3	PKG1	3.7	2.4
p70S6K	3.7	1.7	MAPKAPK3	2.2	1.2	PRKX	2.8	2.3
PKA	3.2	1.5	p70S6K	2.0	1.1	p70S6K	2.8	2.4
PKC	2.6	1.5	IKK	2.0	1.5	Akt2	2.8	2.3
CaMK4	2.4	1.8	Akt2	2.0	1.2	PKG2	2.8	2.3
PRKX	2.0	1.3	JNK2	1.9	1.2	Akt1	2.3	2.3
MAPKAPK2	2.0	1.4	JNK1	1.9	1.2	RSK2	2.2	2.6
IKK	1.9	2.0	JNK3	1.9	1.2	PKD1	2.2	2.7
Pim2	1.9	1.3	RSK2	1.8	1.3	MSK1	2.1	2.6
Akt2	1.8	1.4	MAPKAPK2	1.8	1.1	PKC	2.1	2.2
MAPKAPK3	1.8	1.3	PAK1	1.8	1.3	MAPKAPK3	2.1	2.2
Akt1	1.8	1.3	MSK1	1.8	1.3	Pim2	2.0	2.2
CHK2	1.7	1.4	Akt1	1.7	1.1	CaMK4	2.0	2.4
PFTAIRE1	1.7	1.7	CDK2	1.7	1.2	RAF1	2.0	2.6

Supplementary Figure 3. MTX induces multiple cell signaling pathways in EC independent of prior pro-inflammatory activation with TNFα. HAEC were pre-treated with vehicle (control) or TNFα (0.1ng/ml) for 8h. Media or MTX (100nM) were added to the cells for another 48h (TNFα treatment for total of 56h). Top 15% of kinase hits from STK arrays performed on respectively treated cells (n=3). Final Score indicates combined measure of significance and specificity of permutation analyses. The kinase statistic is computed by the overall change of the peptide set that represent a kinase (value >0 means that kinase is more active in test over control sample).

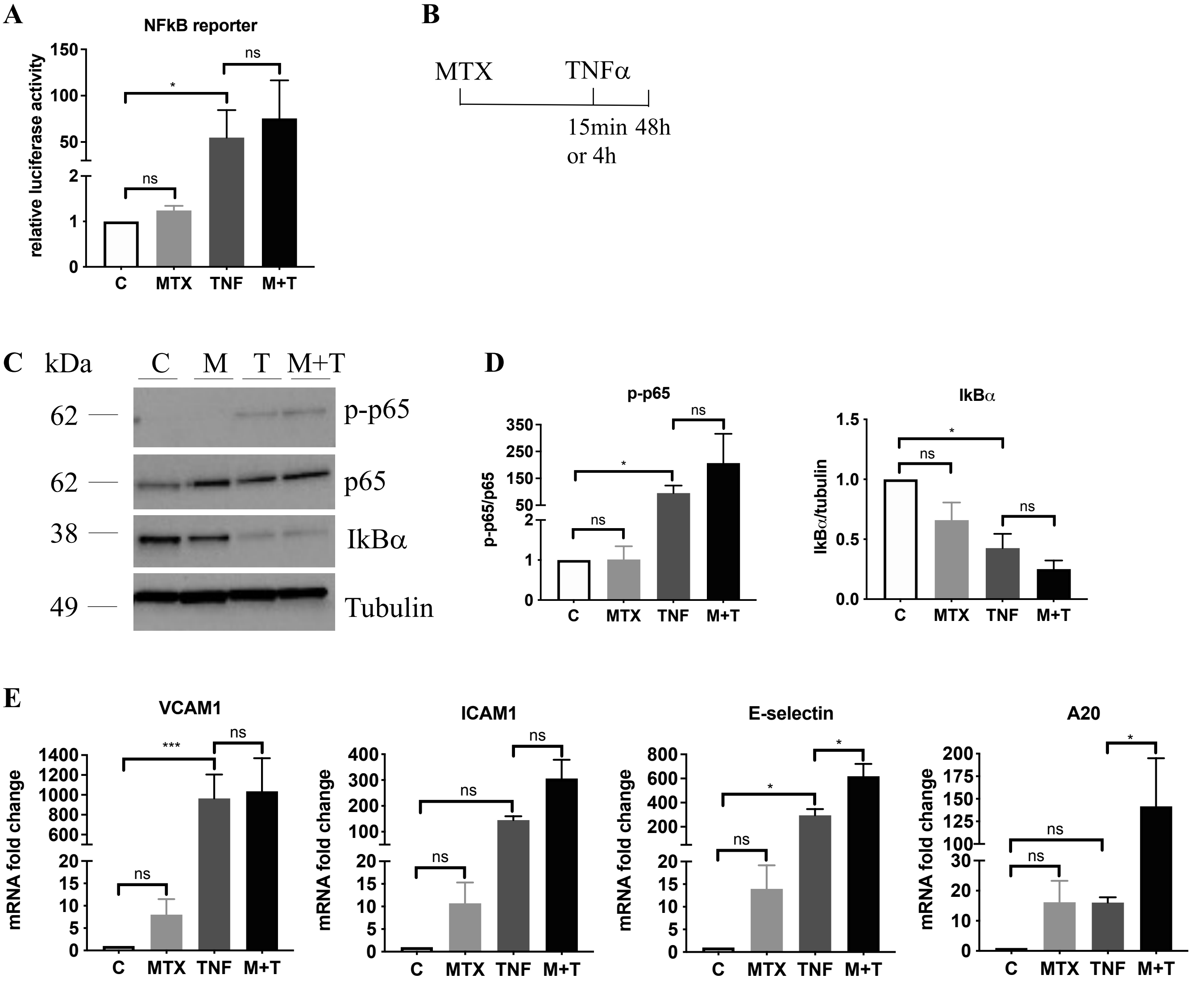

Supplementary Figure 4. MTX does not inhibit TNFα-induced NFκB activation in EC. (**A**) NFκB luciferase reporter assay. HUVEC were transfected with a recombinant adenovirus expressing a NFκB luciferase reporter. Two hours post transfection, cells were treated with media (C, control) or MTX (100nM) for 48h. Media or TNFα (0.1ng/ml) were added for 4h alone or in combination with MTX (M+T). NFκB activity was measured using a Luciferase Reporter Assay System. Data shown as fold change of relative luciferase activity normalized to the control (n=4). (**B-E**) HUVEC were treated with media (C, control) or MTX (M; 100nM) for 48h. Media or TNFα (T; 0.1ng/ml) were added alone or in combination with MTX (M+T) for 15min (cell signaling) or 4h (gene expression) as outlined in (**B**). (**C**) Immunoblot analyses of respectively treated cells. Whole protein lysates were separated by SDS-PAGE. Proteins were detected with antibodies against p-p65 S536 (65kDa), p65 (65kDa), IκBα (39kDa) and α-tubulin (50kDa) (n=3-4). (**D**) Quantification of p-p65 and IκBα protein levels in HUVEC treated as described in (**B**) (n=3-4). (**E**) Gene expression analyses of HUVEC treated as described above by qPCR. Gene expression normalized to GAPDH (n=4). Data were analyzed using a one-way ANOVA and Sidak test for multiple comparisons. Values represent means +/- SEM. ns, not significant. * $p<0.05$. ** $p<0.01$. *** $p<0.001$.

A

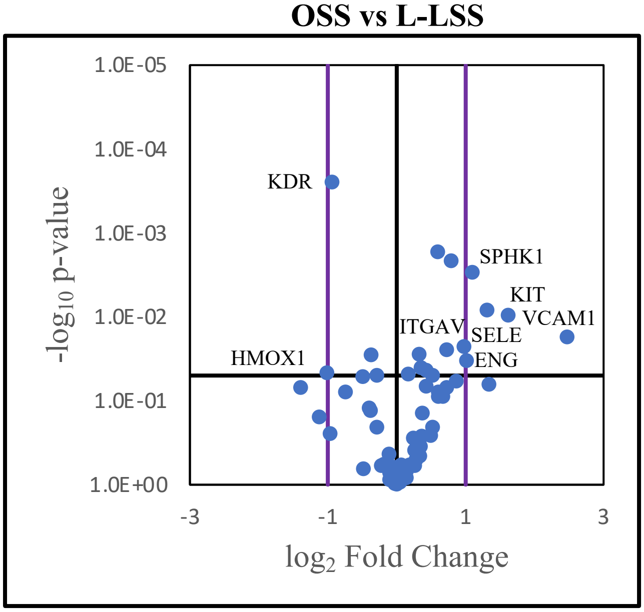

B

Gene	Log_2FC	p-value
KDR	-0.94	0.0002
ANXA5	0.59	0.0017
CCL2	0.79	0.0021
SPHK1	1.09	0.0029
SELE	1.31	0.0082
KIT	1.61	0.0094
VCAM1	2.46	0.0172
ITGAV	0.97	0.0225
SERPINE1	0.72	0.0245
FLT1	0.32	0.0276
TEK	-0.38	0.0283
ENG	1.01	0.0330
TFPI	0.35	0.0400
TGFB1	0.42	0.0430
HMOX1	-1.01	0.0459
PTK2	0.17	0.0474
TNFSF10	-0.29	0.0489
PGF	0.52	0.0490

C

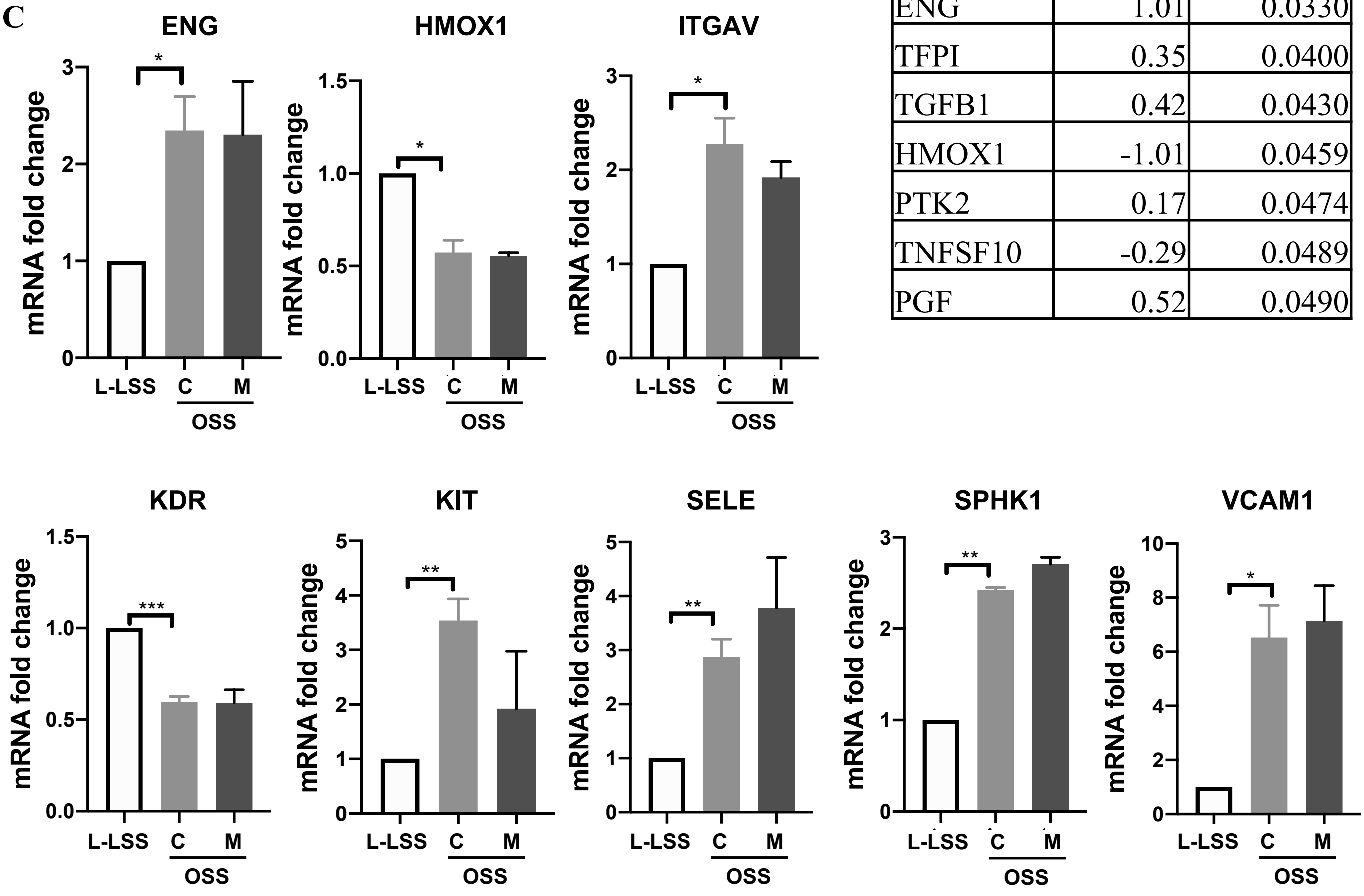

Supplementary Figure 5. OSS-induced gene expression in EC. (A-C) HAEC were subjected to OSS (±5dyn/cm^2, 2Hz) for 48h. Media (control, C) or MTX (M; 100nM) were added to the cells for another 48h under OSS. As control, HAEC were exposed to low LSS (L-LSS; 5dyn/cm^2) for 96h. Gene expression analyses were performed using qPCR arrays (n=3). (**A**) Volcano plot of differentially expressed genes in HAEC subjected to OSS over L-LSS. Purple bars indicate fold change >2; horizontal black bar indicates a p-value <0.05. (**B**) List of significant differentially expressed genes in EC subjected to OSS compared to L-LSS. (**C**) Representative differentially regulated genes in OSS-subjected EC compared to L-LSS. mRNA expression levels normalized to GAPDH and shown relative to low LSS condition. Data were analyzed using unpaired T-tests. Values represent means +/- SEM. ns, not significant. * p<0.05. ** p<0.01. *** p<0.001.

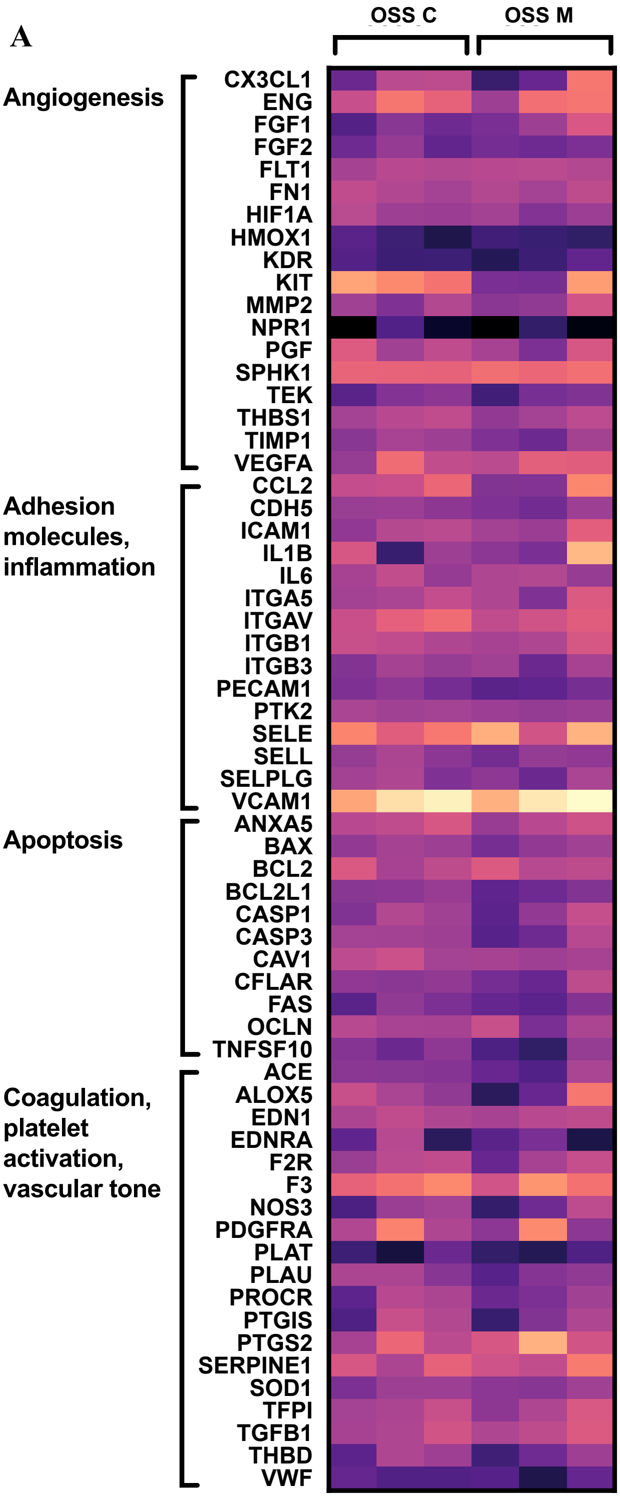

Supplementary Figure 6. MTX does not affect OSS-induced endothelial gene expression. (A) Heat map of log_2FC of genes in EC subjected to OSS with and without MTX compared to L-LSS. HAEC were subjected to OSS (±5dyn/cm^2, 2Hz) for 48h. Media (control, C) or MTX (M; 100nM) were added to the cells for another 48h under OSS. As control, HAEC were exposed to low LSS (L-LSS; 5dyn/cm^2) for 96h. Gene expression analyses were performed using qPCR arrays (n=3).

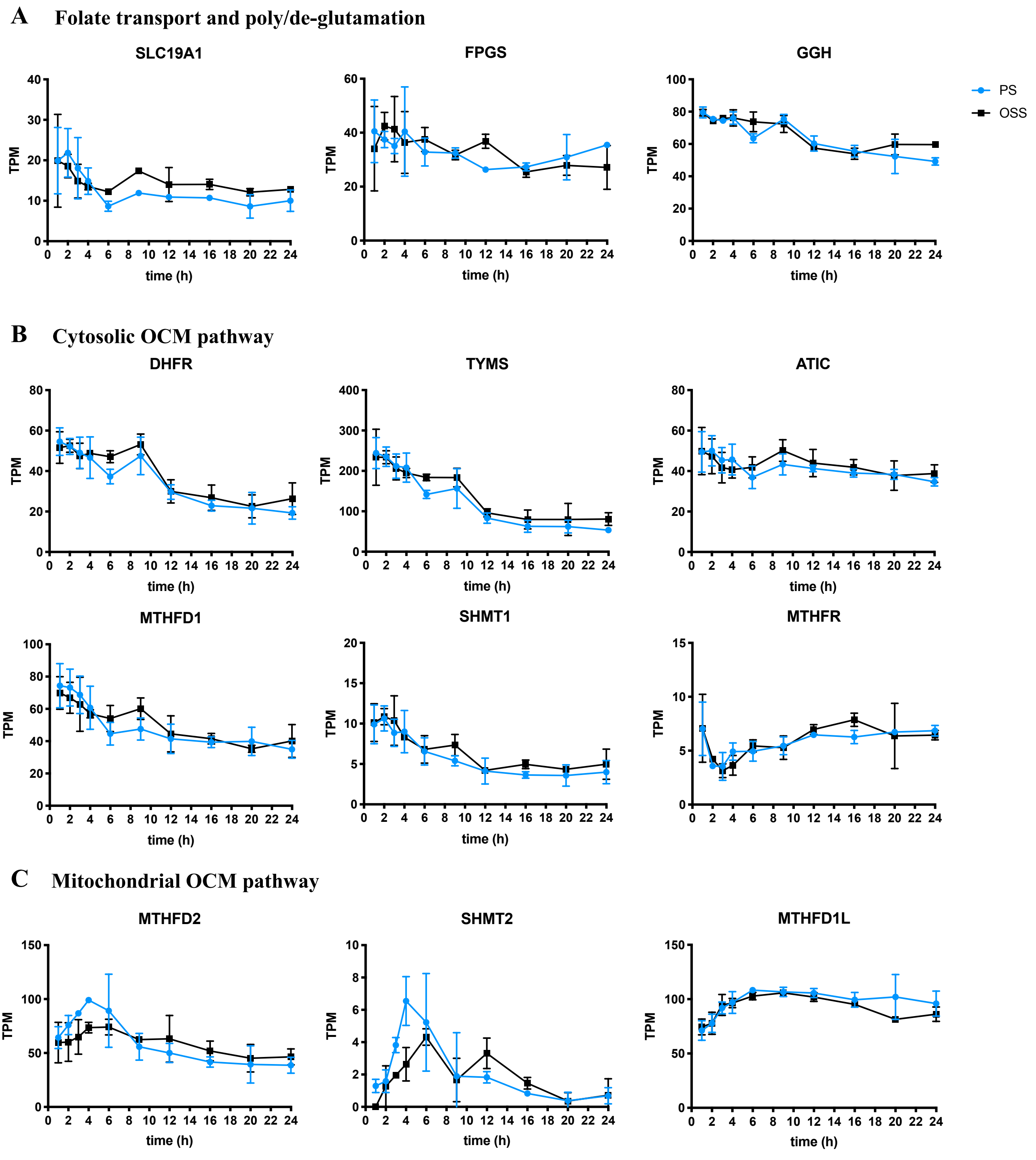

Supplementary Figure 7. Transcript levels of transporters and enzymes related to OCM in HUVEC exposed to shear stress using a published RNA sequencing dataset. (**A-C**) Data obtained from publicly available RNA sequencing dataset (Gene Expression Omnibus (GEO) GSE103672) published by Ajami et al. (32). Transcripts per million (TPM) of selected transporters and enzymes related to OCM in HUVEC subjected to pulsatile (PS, 12±5dyn/cm^2) or oscillatory shear stress (OSS, 0.5±5dyn/cm^2) using a parallel plate model over a period of 24h. (**A**) Proteins involved in cellular folate transport and (de-)polyglutamation. (**B**) Enzymes in cytosolic OCM compartment. (**C**) Enzymes in mitochondrial OCM compartment.

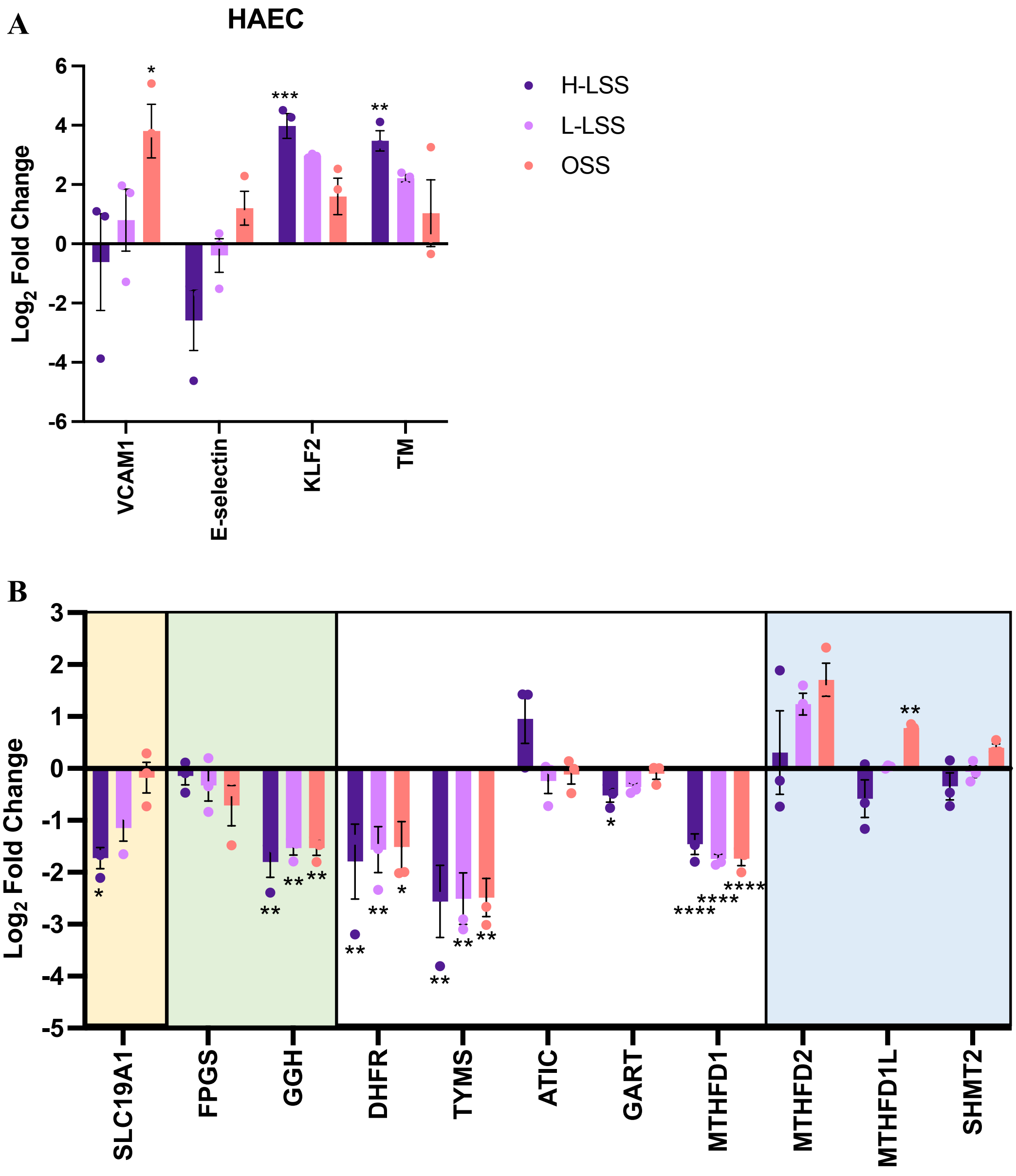

Supplementary Figure 8. Endothelial OCM is also downregulated by shear stress in arterial EC. (**A+B**) HAEC were subjected to static culture (ST), H-LSS, L-LSS or OSS for 48h. (**A**) Log2 fold changes of the mRNA expression of shear stress-related controls (VCAM1, SELE, KLF2 and TM) in HAEC treated as above by qPCR (n=3). Data shown relative to static condition. (**B**) Log2 fold changes of the expression of selected genes related to OCM in HAEC treated as above by qPCR (n=3). Data shown relative to static condition. Proteins involved in cellular folate transport (yellow), (de-)polyglutamation (green), enzymatic reactions in the cytosolic (white) or mitochondrial (blue) compartment. Data were analyzed using a one-way ANOVA and Sidak test for multiple comparisons. Values represent means +/- SEM. ns, not significant. * p<0.05. ** p<0.01. *** p<0.001. **** p<0.0001.

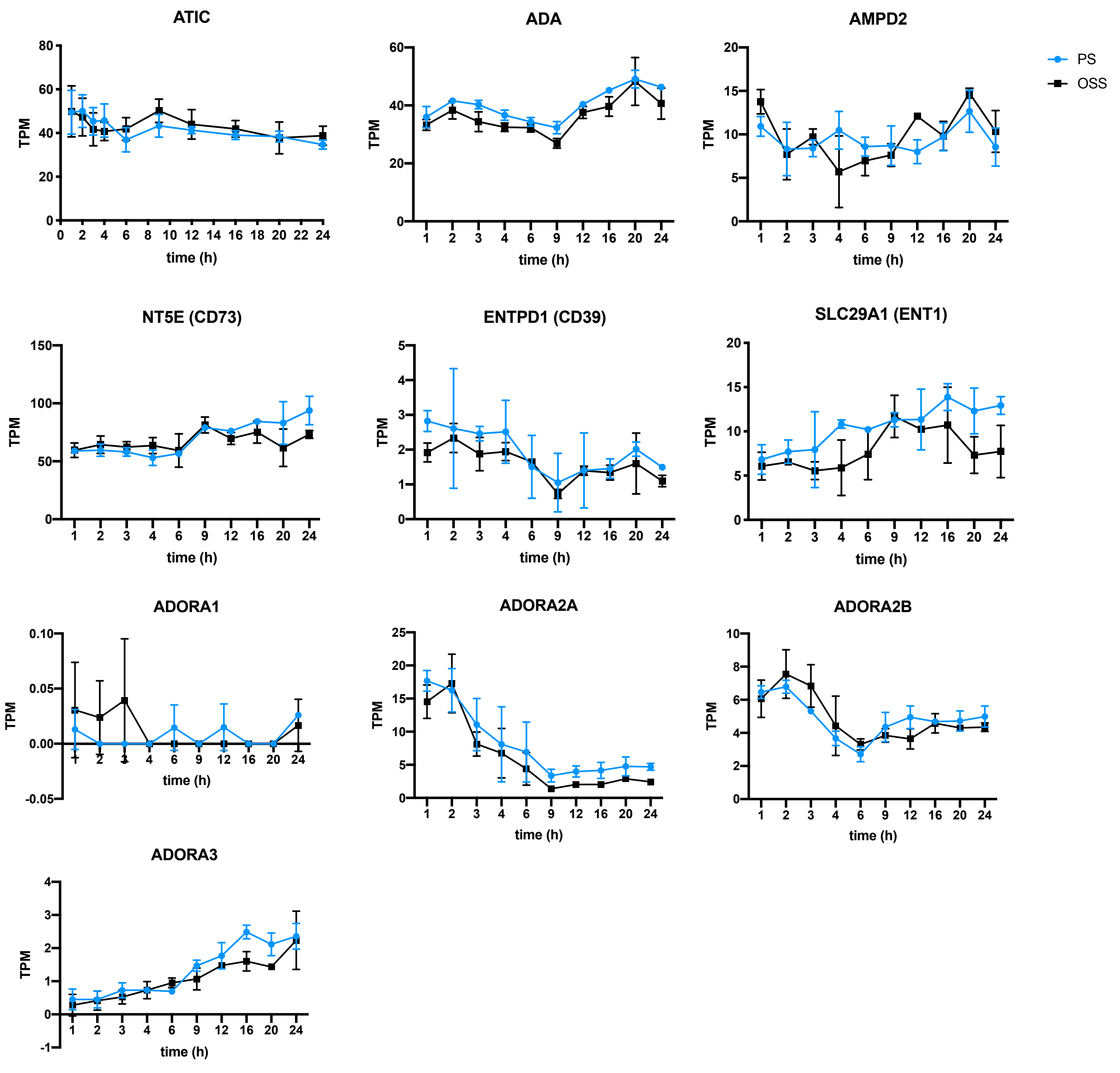

Supplementary Figure 9. Transcript levels of molecules relevant in adenosine metabolism in HUVEC exposed to shear stress using a published RNA sequencing dataset. Data obtained from publicly available RNA sequencing dataset (Gene Expression Omnibus (GEO) GSE103672) published by Ajami et al. (32). Transcripts per million (TPM) of selected transporters and enzymes related to adenosine metabolism and signaling in HUVEC subjected to pulsatile (PS, 12±5dyn/cm^2) or oscillatory shear stress (OSS, 0.5±5dyn/cm^2) using a parallel plate model over a period of 24h.

Figure 1E

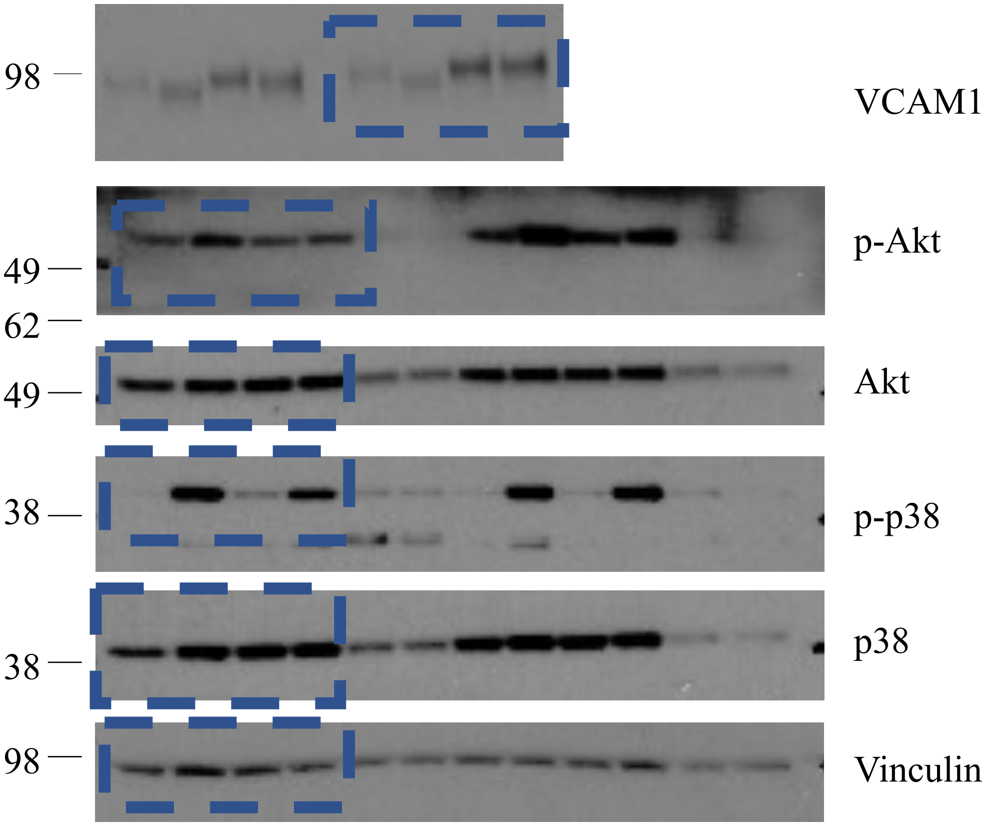

Figure 2A

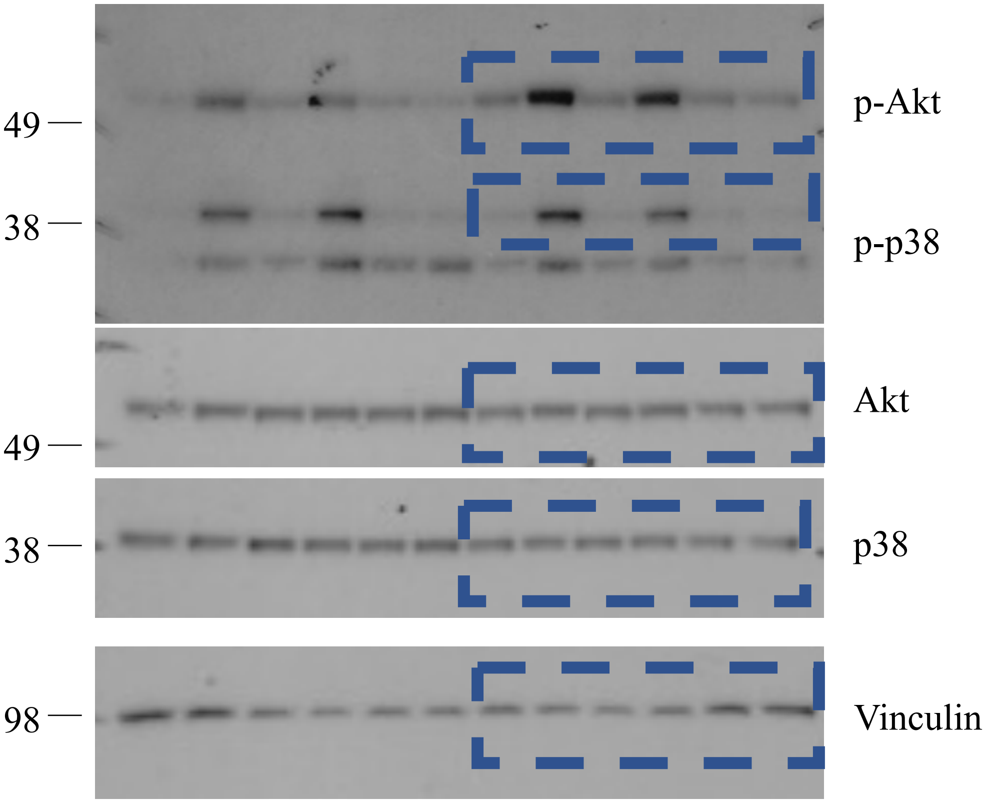

Supplementary Figure 10. Uncropped Western blotting images for figures.

Figure 3A

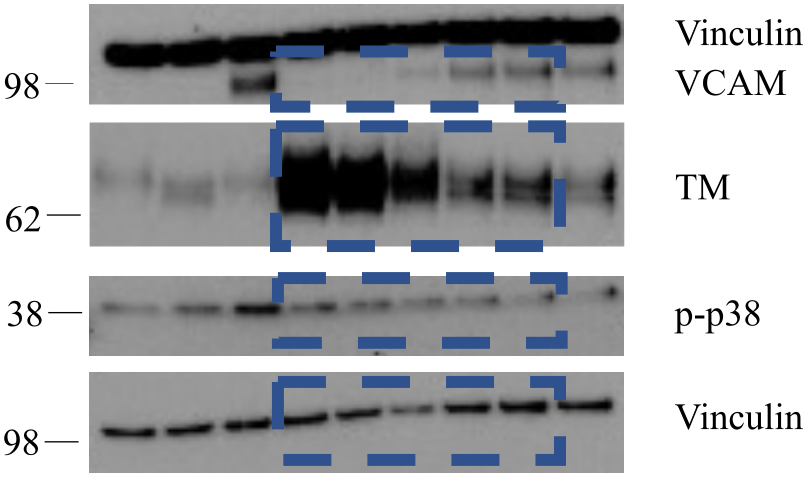

Figure 5C

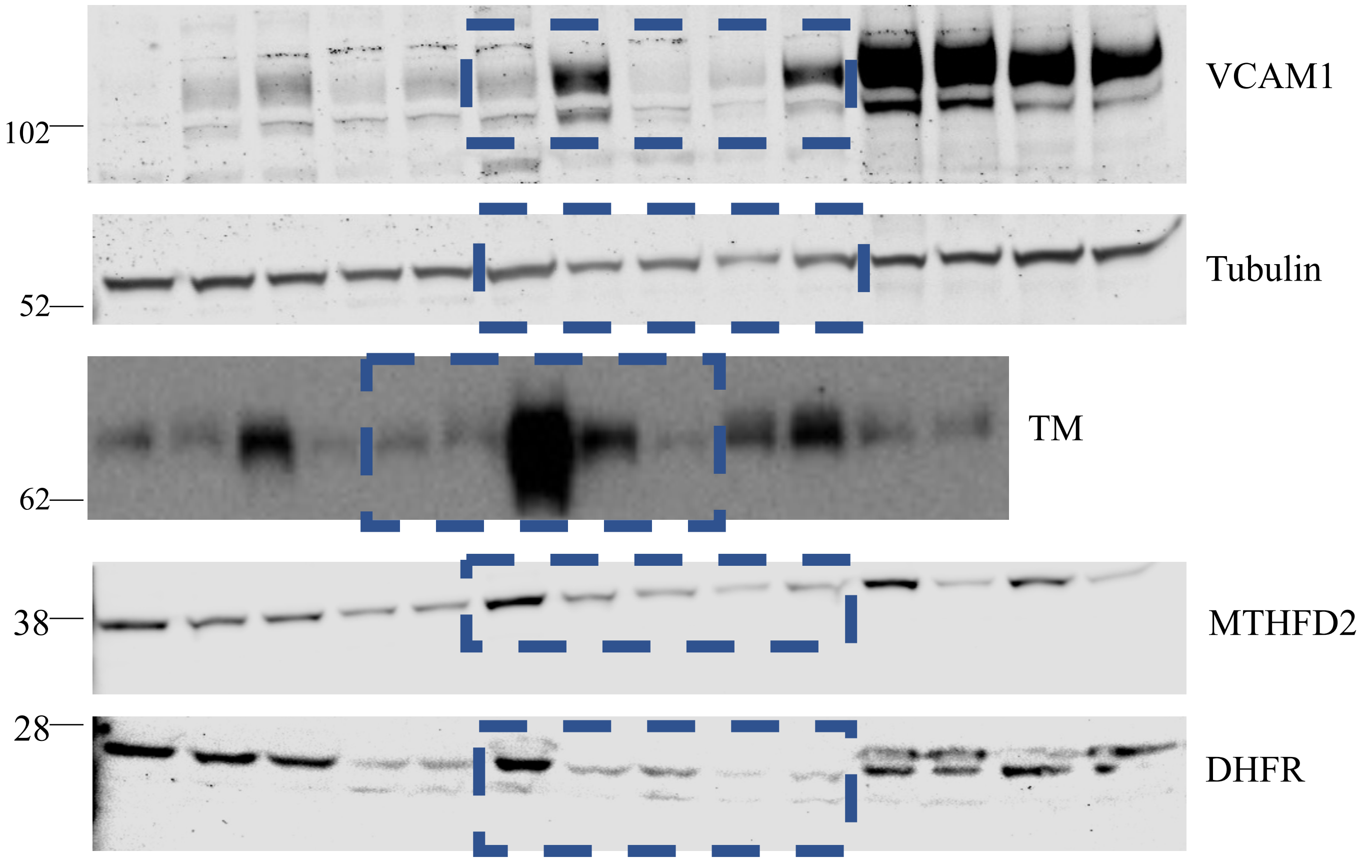

Supplementary Figure 11. Uncropped Western blotting images for figures.

Supplementary Figure 4C

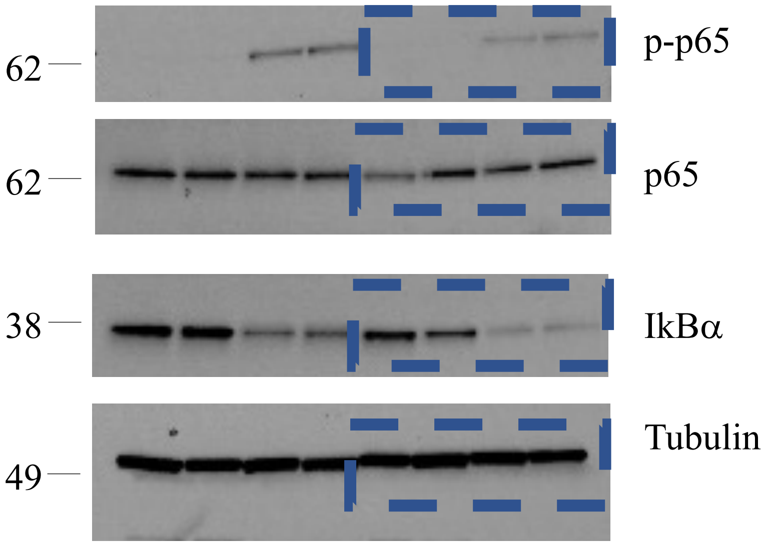

Supplementary Figure 12. Uncropped Western blotting images for figures.
